# Comparative Analysis of Cost, Energy Efficiency, and Environmental Impact of Pulsed Electric Fields and Conventional Thermal Treatment with Integrated Heat Recovery for Fruit Juice Pasteurization

**DOI:** 10.3390/foods14132239

**Published:** 2025-06-25

**Authors:** Giovanni Landi, Miriam Benedetti, Matteo Sforzini, Elham Eslami, Gianpiero Pataro

**Affiliations:** 1 ENEA, Portici Research Center, Piazzale Enrico Fermi, Località Granatello, 80055 Portici, Italy; 2 ENEA, Casaccia Research Center, Via Anguillarese 301, 00123 Rome, Italy; miriam.benedetti@enea.it (M.B.);; 3 Department of Industrial Engineering, University of Salerno, Via Giovanni Paolo II, 132, 84084 Fisciano, Italy; eeslami@unisa.it

**Keywords:** pulsed electric fields, pasteurization, heat treatment, orange juice, cost analysis, environmental impact, energy efficiency

## Abstract

This study evaluates the feasibility of integrating pulsed electric field (PEF) technology with heat recovery for fruit juice pasteurization, comparing it to conventional high-temperature short-time (HTST) pasteurization. Three preheating temperature conditions (35 °C, 45 °C, and 55 °C) and varying heat recovery efficiencies have been assessed to analyze energy consumption, economic feasibility, and environmental impact. The results indicate that, while PEF pasteurization requires a higher initial investment, it improves energy efficiency, leading to significant reductions in utility costs. Across the tested configurations, PEF technology achieved reductions in electricity consumption by up to 20%, fuel gas usage by over 60%, greenhouse gas emissions by approximately 30%, and water consumption by 25%, compared to HTST. The optimal configuration of the PEF process, featuring a 35% waste heat recovery efficiency and a pre-heating temperature of 55 °C, has been identified as the most energy-efficient and sustainable solution, effectively reducing both water consumption and CO_2_ emissions. A life cycle assessment has confirmed these environmental benefits, demonstrating reductions in global warming potential, fossil fuel consumption, and other impact categories. This study suggests that PEF technology can significantly contribute to more sustainable food processing by reducing environmental impacts, optimizing resource usage, and enhancing energy efficiency.

## 1. Introduction

The food and beverage industry incurs substantial operating costs due to its reliance on energy-intensive thermal processes such as pasteurization, sterilization, drying, and cooking [1,2,3]. Energy costs represent a significant portion of total food production expenses, ranging between 20% and 50% [4]. Adopting sustainable and more efficient technologies is essential for reducing energy consumption and decreasing reliance on non-renewable fossil fuels, which significantly contribute to greenhouse gas (GHG) emissions [5,6,7]. Annually, the global food system emits approximately 20 GtCO_2_ equivalent, accounting for 35% of total GHG emissions [8]. Industrial activities within the food sector contribute around 0.5 GtCO_2_ equivalent per year, representing 3.6% of global emissions [9]. Additionally, industrial food production contributes 4.4% to the global water footprint, amounting to 9.1 Gm^3^ per year [10,11].

This is motivating the search for innovative technological solutions that can be easily integrated into existing processing lines, ensuring high product quality and safety while enhancing the economic and environmental sustainability of the food industry.

In this frame, non-thermal technologies, such as pulsed electric fields (PEFs), have gained attention as alternatives or complement the traditional thermal preservation methods of liquid foods [12]. In PEF processing, the liquid food product is passed through a continuous flow treatment chamber equipped with metal electrodes and exposed to repetitive very short (1–10 µs) and intense (10–40 kV/cm) electric pulses provided by a pulse generator [13]. Several studies have demonstrated that PEF treatment induces electroporation in the membranes of bacterial and yeast cells, effectively reducing microbial load. The degree of inactivation depends on factors such as field strength, energy input, temperature, microbial strain, and the properties of the food matrix [13,14,15,16,17]. Furthermore, due to its gentle, low-temperature processing, PEF preserves food quality, maintaining nutritional value, freshness, and color [18,19].

Moreover, the continuous flow operability of PEFs allows for very short processing times and easy integration into existing processing lines. Recent advancements in chamber geometry, like co-field designs, and high-pulsed power systems have supported its scale-up from lab to industrial use [20,21,22,23].

Nevertheless, commercial-scale applications for pasteurizing liquid foods, including fruit juices and smoothies, remain limited [20]. The primary barriers to adopting PEF technology for liquid food preservation in the beverage industry are the significant initial investment and high processing costs [20,24]. Energy efficiency is key to the commercial adoption of PEF processes as an alternative to heat-based preservation methods [17,25,26,27]. To address this, a hurdle approach combining PEF technology with moderate heating has been proposed, aiming to reduce costs while maintaining effective pasteurization. This combined strategy has shown a synergistic effect, enhancing microbial inactivation and further improving energy efficiency [15,19,28,29]. Additionally, operating at higher temperatures allows the recovery of the electrical energy supplied, which can be reused via a heat exchanger to preheat the product, enhancing process efficiency [30].

However, there is limited scientific literature that systematically analyzes costs and energy efficiency, especially in relation to varying preheating temperatures and thermal recovery strategies during PEF treatment. Furthermore, research on the environmental impact of commercial PEF food processing remains scarce [24,31,32].

The few existing studies are outdated and need to be revised to reflect recent advancements in technology and the growing number of PEF manufacturers [20]. For example, Sampedro et al. compared the costs of orange juice pasteurization using conventional thermal methods and PEFs under fixed operating conditions (30 kV/cm and 60 °C) [24,32]. A more recent study further evaluated the cost, energy efficiency, and environmental impact of both methods, considering heat recovery rates of 0–40%. In this analysis, PEFs were applied at 36 kV/cm, 90 kJ/kg energy input, and 40 °C preheating [33].

Therefore, a comprehensive and up-to-date analysis of the cost and environmental impact of PEF pasteurization is urgently needed by commercial juice processors. This analysis should account for heat recovery and integration with moderate heating across a broad range of parameters to enhance energy efficiency. Within this context, optimizing PEF parameters to achieve the required 5 Log microbial reduction [34,35], while minimizing energy consumption, is essential for reliable cost and sustainability assessments. Additionally, scaling up lab-optimized conditions to industrial levels is essential, but current literature offers limited guidance, especially regarding process capacity effects.

This study seeks to address the existing knowledge gap by providing a comprehensive analysis of the costs, energy efficiency, and environmental impacts associated with large-scale pasteurization of orange juice using PEF processing, compared to the traditional high-temperature short-time (HTST) thermal method. Optimal processing conditions for PEF pasteurization, combined with mild heating, have been identified through an extensive review of the literature. A detailed cost model has been developed to evaluate the economic viability of implementing a commercial-scale PEF system under various heating conditions and thermal recovery efficiencies. Key economic indicators, including net present value (NPV) and payback period (PBP), have been used to pinpoint the most favorable configurations across diverse energy scenarios, enabling a thorough and efficient assessment of economic feasibility. Furthermore, the study incorporates a life cycle assessment (LCA) to examine the environmental performance of both conventional and innovative pasteurization methods. This includes an analysis of the impact on various midpoint environmental indicators, providing a holistic perspective on the trade-offs between these technologies.

## 2. Materials and Methods

### 2.1. PEF Operating Conditions

In this work, the optimal PEF process parameters were derived from the study published by Toepfl [36]. According to the literature survey, this is the only study that focuses on identifying suitable processing and equipment parameters for scaling up PEF processing in liquid food pasteurization, including fruit juices. Specifically, PEF systems with different average power and treatment capacities, such as 20 L/h (3 kW), 200 L/h (5 kW), and 2000 L/h (30 kW), were developed to evaluate the scalability of the PEF technique for liquid food pasteurization. The study found that solid-state setups are suitable for long-term liquid treatment. The co-linear chamber with a static mixer showed the highest efficiency, and titanium electrodes offered better corrosion resistance than stainless steel.

The 3 kW PEF system was utilized for lab-scale experiments assessing microbial inactivation. These experiments evaluated the combined effect of PEF and mild heating on the inactivation of pathogenic and spoilage microorganisms, including *Escherichia coli* (Gram-negative bacteria), *Bacillus megaterium* (Gram-positive bacteria), and *Saccharomyces cerevisiae* (yeast), across a range of liquid food matrices such as orange juice.

The experiments were conducted by varying the inlet temperature (35 °C, 45 °C, and 55 °C) in the co-field treatment chamber while maintaining a constant electric field strength (E = 20 kV/cm) and flow rate (20 L/h). At each inlet temperature, the total specific energy input (*W_PEF_*) was set between 5 and 120 kJ/kg by varying the pulse repetition rate (*f*). The total specific energy input can be calculated by Equation (1) [27],

(1) $$W_{PEF}=f\frac{v}{\dot{m}}\int_{0}^{\infty }\sigma \left(T\right)E{\left(t\right)}^{2}dt$$

where *σ*(*T*) denotes the electrical conductivity of the treatment of the liquid food (in S/cm), *E* is the electric field strength (in kV/cm), *ν* is the volume of the treatment zone (in m^3^), and $\dot{m}$ is the mass flow rate (in kg/s), respectively.

Post-treatment, survivors were enumerated to determine the optimal *W_PEF_*_,*opt*_ values for each inlet temperature, representing the minimum specific energy input required to achieve a 5 Log-reduction in microbial load (see Table S1 of the Supplementary Materials). The study also included sensory analysis and shelf-life evaluation of orange juice pasteurized using conventional thermal methods and PEF treatment.

Finally, to assess the impact of process capacity on microbial inactivation levels, the optimal processing conditions for juice pasteurization identified in the lab-scale PEF experiments (20 L/h, 3 kW) were tested at larger scales of 200 L/h (5 kW) and 2000 L/h (30 kW).

### 2.2. Model Description

#### 2.2.1. Systems Description for the Pasteurization Process of Fruit Juice

**Figure 1:**
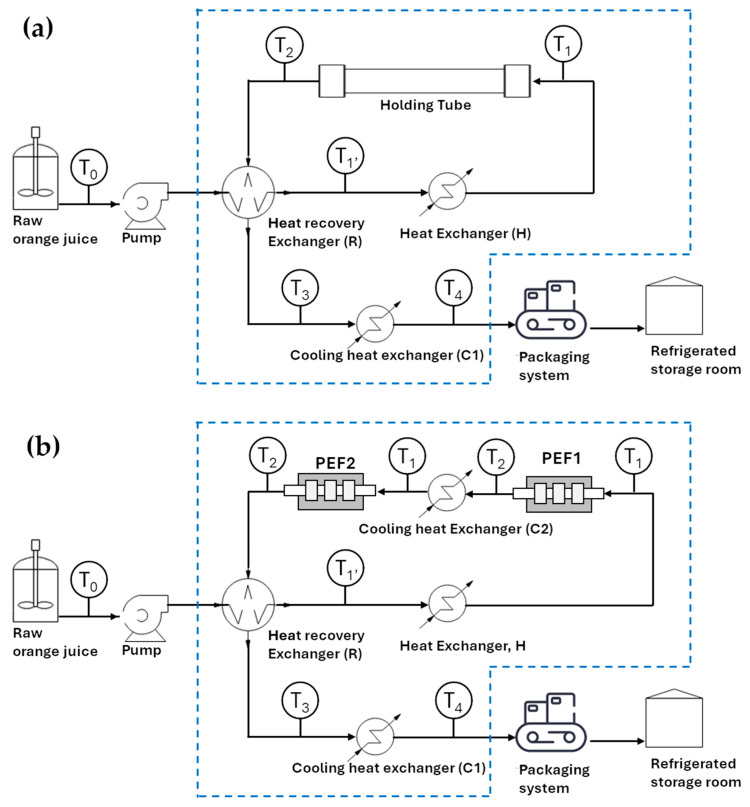
Schematic diagrams of designed commercial (**a**) thermal HTST and (**b**) PEF system for the pasteurization of orange juice. The blue dashed line represents the system boundaries for the life cycle assessment described in Section 2.3.2.

Commercial-scale pasteurization processes of fruit juice based on conventional and unconventional technologies were comparatively analyzed. Figure 1a shows a simplified schematic of the conventional thermal HTST pasteurization treatment for orange juice. This process operates at 90 °C (*T*_1_) for 15 s and includes a heat recovery stage as a strategy for energy-saving [37]. In contrast, Figure 1b illustrates the PEF pasteurization process, which involves preheating the raw juice to specific inlet temperatures (*T*_1_) (35 °C, 45 °C, or 55 °C) before undergoing PEF treatment, followed by a heat recovery step. The PEF treatment is performed in two consecutive chamber modules (PEF1 and PEF2), with an intermediate cooling phase between them. Each module consists of two co-linear chambers. The PEF pasteurization process was optimized for energy input (*W_PEF_*_,*opt*_) at each inlet temperature using data from Toepfl [36], as outlined in the previous section. Notably, PEF treatment causes a temperature rise due to the ohmic heating phenomenon, which generally enhances treatment effectiveness and facilitates further heat recovery [24,30,38]. Equation (2) was employed to estimate the temperature increase (∆T) of the juice in each module of the PEF treatment chamber (Table S1). This calculation was based on the optimal total specific energy input ${(W}_{PEF,opt},\;in\;kJ/kg)$, assuming adiabatic conditions for each chamber, except for a 5% heat loss through the metal electrodes [39]. Therefore, ∆T can be expressed as

(2) $$∆T=\frac{1}{2}\cdot \frac{W_{PEF,opt}}{C_{ps}}\cdot 0.95$$

where 2 indicates the number of chamber modules through which the total specific energy ${\;(W}_{PEF,opt})$ is applied and *C_ps_* is the specific heat capacity of orange juice, assumed to be 3.89 kJ/kg°C [35]. As a result, the temperature (*T*_2_) of the juice exiting each module of the PEF chamber is equal to *T*_1_ and increased by Δ*T*. To maintain the inlet temperature at T_1_ for the juice entering the second module of the PEF chamber, a cooling heat exchanger (C2) was employed, using cold water supplied by a chiller.

Regardless of the pasteurization treatment, the incoming fresh juice at a temperature of *T*_0_ (4 °C) was first preheated by the hot juice exiting the thermal or PEF pasteurization unit. This preheating occurred in a heat recovery exchanger (***R***), raising the juice temperature to a given value *T*_1’_ dependent on a waste heat recovery (WHR) percentage of 25–65% from the pasteurized juice [40]. The WHR percentage was calculated using Equation (3), considering heat exchange between two juice streams of the same type and mass flow rates and assuming nearly identical specific heat capacities (3.89 kJ/kg°C) [1,32].

(3) $$WHR=100\times \frac{\left(T_{1\prime }-T_{0}\right)}{\left(T_{1}-T_{0}\right)}$$

where *T*_2_ is the temperature of the pasteurized juice and *T*_3_ is the temperature of the pasteurized juice after it exits the heat recovery stage.

The juice is then heated to the final temperature *T*_1_ (90 °C for HTST, and 35, 45, or 55 °C for PEF) in a second heat exchanger (*H*) using saturated steam from a boiler. Following this, the juice undergoes either a 15 s holding phase for HTST [37] or PEF treatment at a field strength (*E*) of 20 kV/cm, with the optimal total specific energy input (*W_PEF,opt_*) for each inlet temperature (35, 45, and 55 °C).

Both HTST and PEF treatment conditions were selected to achieve a 5-log reduction in the microbial population, as required by the US Food and Drug Administration (FDA) [34]. The pasteurized juice, exiting the heat recovery exchanger at temperature T_1_, is subsequently cooled in a final cooling heat exchanger (C1). This unit uses chilled water at an inlet temperature of 2 °C, supplied by a chiller, to lower the juice temperature to 7 °C (*T*_4_) prior to packaging and storage. It is important to note that energy losses in the thermal balance of each heat exchanger in both PEF and HTST processes were not considered in this study. The detailed designs and heat transfer calculations for each heat exchanger shown in Figure 1 are provided in the Supplementary Materials (Figure S1 and Tables S2–S9) [40,41].

#### 2.2.2. Cost Analysis and Energetic Scenario

A cost model was developed to comparatively evaluate the costs associated with conventional HTST and heat-assisted PEF pasteurization processes (Figure 1) within a medium-sized facility producing pasteurized orange juice at the same throughput. To match the treatment capacity used for validating optimal PEF conditions (see Table S1) and to facilitate comparison with previous studies [24], the facility was assumed to process 16,500,000 L of orange juice annually. This was accomplished with a throughput of 3000 L/h, operating 20 h per day, with 4 h each day allocated for equipment sanitation and maintenance. This schedule resulted in a total operational duration of 5500 h/year, equivalent to 275 working days. The comparison between the costs of traditional HTST and PEF pasteurization was conducted for the same heat recovery rates ranging from 0% to 65%. Additionally, the impact of combining PEF with preheating the juice at moderate temperatures (35 °C, 45 °C, and 55 °C) was also evaluated.

The models were developed using Excel (Microsoft 365) to determine the sizing of unit operations, utility consumption, and both capital and unit pasteurizing costs. These models were constructed based on data collected from equipment manufacturers and relevant scientific literature [15,16,24,36]. Information regarding the cost and energy consumption of all ancillary units, such as pumps, heat exchangers, steam-generating boilers, and chilling units, was integrated into the model as inputs corresponding to the desired production scale.

Subsequently, the unitary production cost of juice was calculated as the output of the model. The electricity costs used in this study were derived from EUROSTAT data, representing average industrial electrical rates for the first semester of 2023 [42]. These rates ranged from a minimum of EUR 0.25/kWh to a maximum of EUR 0.35/kWh. Steam charges were determined based on 2023 EUROSTAT data, reflecting the cost of steam generated in a natural gas boiler, ranging from EUR 0.6/smc to EUR 1.5/smc when delivered to an industrial customer [42]. The cost of water was EUR 3.5/m^3^, an average rate in Europe [43]. To strengthen the economic model, a sensitivity analysis was conducted by varying electricity and gas prices within these ranges, thus capturing potential market fluctuations and increasing the reliability of cost estimates. It is noteworthy that the energy estimates in this study are specific to the European Union and the processing of fruit juice. Energy costs may vary by country, region, energy source, and food product, leading to differing absolute cost estimates for energy.

The cost estimates in this work do not include juice storage or other upstream and downstream processes related to the pasteurization unit, including juice packaging. Additionally, costs for raw materials and waste treatment expenses were not factored into the model. Capital costs also excluded charges for common facilities, utilities, offices, environmental controls, land acquisition, site development, working capital, and construction-related expenses. These costs are variable and site-specific, typically addressed during the later engineering stages of a project [24].

To estimate annual costs related to capital investment, a plant life of 10 years was assumed. Depreciation was set at 10% of the capital cost per year, with maintenance and administrative expenses calculated at 3% and 2% annually, respectively, to account for upkeep, repairs, and operational overhead. The total annual costs associated with the capital investment were determined by summing these values, including depreciation, maintenance costs, and administrative charges. The cost estimates included in the model were used to evaluate two economic indicators: net present value (NPV) and payback period (PBP). These indicators were essential for effectively comparing the economic sustainability of conventional pasteurization methods with innovative PEF pasteurization processes.

The NPV, a financial metric used to assess the profitability of an investment by considering the time value of money, was calculated as the sum of discounted cash flows over the investment period, minus the initial cost C_0_ [44].

(4) $$NPV=\sum_{t=0}^{N}\frac{{CF}_{t}}{{\left(1+r\right)}^{t}}-C_{0},$$

where ${CF}_{t}$ represents the net cash flow at time t discounted at the rate r (set at 10%), *N* is the number of years, and $C_{0}$ denotes the initial cost of the investment. A positive NPV indicates that the investment is expected to generate more value than its cost, while a negative NPV suggests the opposite.

The PBP measures the time required for an investment to recover its initial cost through the cash flows it generates. It can be calculated according to Equation (5) [44],

(5) $$PBP=t^{*}+\frac{D}{{CF}_{t}^{*}}$$

where $t^{*}$ represents the last complete year before the total recovery of the initial investment, D denotes the fraction of the additional year needed to recover the initial investment beyond the last complete year (determined by linear interpolation), and ${CF}_{t}^{*}$ signifies the net cash flow in the last complete year before total recovery, discounted at the rate r equal to 10%. Lower values of the PBP denote a shorter time period required for the investment to recover its initial cost, suggesting higher financial efficiency and attractiveness.

### 2.3. Environmental Impact Analysis

#### 2.3.1. Greenhouse Gas GHG Emissions Assessment

The environmental impact of a process is often evaluated by assessing its greenhouse gas emissions, typically expressed in carbon dioxide equivalents (CO_2-eq_) [31,42,45]. In this study, the environmental impact of PEF and thermal pasteurization processes was estimated by calculating the overall CO_2_-equivalent emissions (in kilograms per hour) resulting from electricity and natural gas consumption, using specific conversion factors [31,42]. For electricity, the conversion factor used was 0.285 kg CO_2-eq_ per kWh, calculated using an activity-based approach [42]. This factor accounts for the total lifecycle emissions associated with the production and transmission of electricity, including upstream emissions from resource extraction and transportation. For natural gas, the emission factor utilized was 2 kg CO_2_-eq per standard cubic meter (smc) of gas consumed [42]. This factor reflects the CO_2_-equivalent emissions generated per unit of natural gas used in the process. It is important to note that these conversion factors can vary depending on the geographical area and the specific type of fuel used in electricity generation and natural gas extraction.

#### 2.3.2. Life Cycle Assessment

Life Cycle Assessment is a comprehensive accepted methodology used to assess the environmental impacts of a product or service throughout all stages of its life cycle. ISO 14040 and 14044 frameworks (2006) provide a structured approach for quantifying, analyzing, and interpreting environmental effects across all stages of a product’s life cycle. [46,47]. According to these standards, the principles and framework for LCA comprise key phases of goal and scope definition, life cycle inventory (LCI) analysis, life cycle impact assessment (LCIA), and finally life cycle interpretation [46,47].

Defining the goal and scope of an LCA study is a pivotal first step that establishes the purpose of the analysis and delineates the system boundaries. This phase ensures the inclusion of all relevant stages and processes within the life cycle of a product, providing results that are reliable, consistent, and suitable for informed decision-making [48]. This study aims to evaluate the environmental impact of innovative technologies in reducing the environmental footprint during the pasteurization phase of orange juice production. Specifically, it compares the environmental effects of two pasteurization methods: conventional HTST and heat-assisted PEF pasteurization. The system boundary is “gate-to-gate”, focusing solely on the pasteurization stage. The analysis considers the consumption of water, natural gas, and electrical energy during pasteurization for both methods, all within the defined boundaries, as illustrated in Figure 1.

In LCA, the functional unit (FU) serves as a standardized measure of the performance of the product, service, or process under analysis [47]. It provides a consistent reference for inventory data, enabling meaningful comparisons between different products or processes. In the food industry, the FU is commonly defined based on the unitary mass or volume of the target product, with all associated inputs and outputs calculated accordingly [48]. For this study, the FU is defined as one liter of orange juice pasteurized under optimized conditions for both conventional and novel methods.

The life cycle inventory (LCI) phase is a crucial step in LCA, involving the quantification of all inputs and outputs across the entire life cycle of a product. The accuracy and reliability of an LCA study largely depend on the quality of the data collected during this phase [49]. In this study, data on water, electricity, and methane consumption during the pasteurization phase were obtained from a combination of equipment manufacturers and existing literature. This data were normalized to represent resource consumption for the pasteurization of one liter of orange juice using both HTST and heat-assisted PEF methods, with additional details provided in Table S10 of the Supplementary Materials.

The background data for this study, including information on the production of electricity, methane, and water, were sourced from the Ecoinvent database (V3.5). While the data primarily reflects the Italian context where available, in cases where country-specific information was lacking, the study relies on data that are representative of the broader European context.

The environmental impact associated with producing 1 L of pasteurized orange juice was assessed using the CML-IA baseline method (V3.05) within the SimaPro software (9.0.0.48), developed by the Center for Environmental Science (CML) at Leiden University [50]. This Life Cycle Assessment methodology evaluates 11 midpoint impact categories: Global Warming Potential (GWP100a), Abiotic Depletion (AD), Abiotic Depletion from fossil fuels (ADf), Ozone Layer Depletion (ODP), Human Toxicity (HT), Acidification Potential (AP), Eutrophication Potential (EP), Freshwater Aquatic Ecotoxicity (FAET), Marine Aquatic Ecotoxicity (MAET), Terrestrial Ecotoxicity (TET), and Photochemical Oxidation (PO). These indicators were employed to assess the environmental impacts of both conventional and innovative pasteurization processes for orange juice.

## 3. Results

### 3.1. Effect of Combined PEF–Moderate Heating on Microbial Inactivation

This study builds on Toepfl’s [36] previous work on microbial inactivation in orange juice, which investigated the effects of moderate heat combined with PEF treatment across equipment capacities ranging from 20 to 2000 L/h. The goal is to identify the optimal PEF processing conditions, validated at a large scale, to achieve the required pasteurization standard (5 log-cycle reduction) [34] while minimizing electrical energy consumption and improving process sustainability. These findings will serve as a baseline for comparing HTST and PEF pasteurization from both economic and environmental perspectives.

As reported by Toepfl, PEF treatment at 20 kV/cm effectively inactivated up to 6 log-cycles of *E. coli*, *Bacillus megaterium*, and *S. cerevisiae* in orange juice. Inactivation increased with higher inlet temperatures (35–55 °C) and energy inputs (10–120 kJ/kg), varying by microbial strain [36].

Notably, among the tested microorganisms, *E. coli* was both the most resistant to PEF treatment and the only pathogenic microorganism that must be specifically targeted and inactivated to ensure a minimum 5-log reduction, as required by FDA guidelines [34], due to its potential to cause serious foodborne illnesses. In contrast, *S. cerevisiae* and *Bacillus megaterium*, while commonly found in juices as part of the natural microbial flora, are not classified as pathogens in this context and are therefore not primary targets for pasteurization from a safety perspective. Consequently, this study focuses on identifying the PEF conditions required to achieve a 5-log (99.999%) reduction in *E. coli*, a key requirement for obtaining FDA approval for juice pasteurization processes.

The results achieved by Toepfl [36] also demonstrated a clear synergistic effect between inlet temperature and PEF treatment on microbial inactivation. At a constant energy input of 60 kJ/kg, increasing the inlet temperature from 35 °C to 55 °C enhanced *E. coli* reduction from 2.0 to 5.8 log-cycles. This synergy between heat and PEF aligns with findings from earlier studies on microbial inactivation in various liquid food matrices [15,19,24,25,51].

Interestingly, in all these studies, it was suggested that mild heating of the microbial suspension helps make the lipid bilayer of the cell membrane more susceptible to breakdown when exposed to an external electric field [15,28]. This mechanism may also explain the synergistic effect of combined PEF and heat treatment observed in the literature, particularly in the reduction of *E. coli* populations [36].

From an energy efficiency standpoint, Toepfl [36] showed that higher inlet temperatures significantly lowered the energy required for effective PEF treatment. To achieve a 5 log-cycle reduction, the optimal energy inputs (*W_PEF,opt_*) were 102, 79, and 38 kJ/kg at inlet temperatures of 35 °C, 45 °C, and 55 °C, respectively. These values represent the minimum energy needed for pasteurization, as detailed in Table S1 of the Supplementary Materials.

This finding is consistent with previous studies, which suggest that less PEF energy is required for inactivation at higher temperatures [15,25,27]. For instance, research has shown that increasing the treatment temperature from ambient to between 35 °C and 55 °C reduces the electrical energy needed to achieve a 6 log-cycle reduction of *E. coli* in apple juice, from over 100 kJ/kg to just 40 kJ/kg when the initial temperature is 55 °C [25].

However, since electric energy is dissipated into the liquid media, this results in a temperature increase due to the Joule effect during the treatment. The temperature increase, calculated using Equation (2) corresponding to *W_PEF,opt_*, is reported in Table S1. The results show that applying PEF treatment at the minimum optimal energy levels required for a 5 log-cycle reduction at inlet temperatures of 35 °C, 45 °C, and 55 °C resulted in temperature increases of 12.5 °C, 9.6 °C, and 4.6 °C, respectively. Despite reaching a maximum temperature of 59.6 °C, the process remained within non-lethal thermal conditions and was carried out over a very short duration.

Utilizing a combined heat and PEF treatment not only reduces equipment and operational costs but also potentially enhances product quality due to the milder processing conditions compared to conventional thermal pasteurization [52,53,54]. In this regard, Toepfl found through sensory analysis that, compared to freshly squeezed juice, a detectable cooked flavor developed when the maximum temperature exceeded 61 °C [36]. However, this temperature was never reached under the optimal processing conditions required to achieve a 5-log reduction of *E. coli*. Moreover, after treatment under these optimal PEF conditions, the author found that the shelf life of orange juice increased from 7 days (untreated) to over 21 days, while maintaining fresh-like taste and product quality.

Finally, the comparison of different treatment capacities revealed that the level of inactivation was not dependent on the flow rate, within a range of 20 to 2000 L/h, provided that the energy input and flow pattern were constant. This demonstrated a successful transfer of processing conditions from the laboratory to the industrial scale.

Based on these findings, the processing conditions for the cost analysis of large-scale orange juice pasteurization using combined PEF and moderate heating were selected in alignment with the study of Toepfl [36], which achieved a 5-log reduction in microbial load at each inlet temperature, as summarized in Table S1.

### 3.2. Evaluation of Energy and Water Consumption and Environmental Impact in the Pasteurization Process

#### 3.2.1. HTST Process

Table 1 shows the temperature of the fresh juice leaving the heat recovery stage (R) $T_{1^{\prime }}\;$(see Figure 1a), along with the residual thermal power $Q_{H,HTST}$ supplied to the juice in the subsequent heating stage (H) to reach the pasteurization temperature *T*_1_, and the related steam consumption $S_{H1},$ as a function of the waste heat recovery (WHR) efficiency ranged between 0 to 65%.

**Figure 2:**
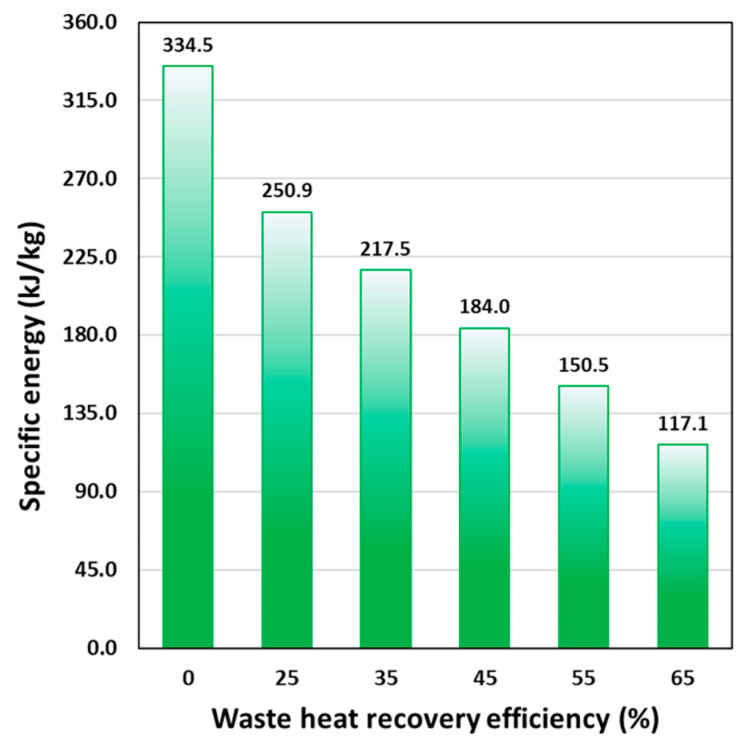
Specific energy consumption ($W_{th,HTST})$ for the orange juice pasteurization process at T_1_ = 90 °C using HTST treatment, with varying waste heat recovery efficiency.

As expected, the increase of the WHR efficiency contributes to elevating the temperature of raw juice from its initial value of 4 °C up to $T_{1^{\prime }}$. Consequently, this augmentation corresponds to a reduction in the required thermal power $Q_{H,\;HTST}$ to attain the target process temperature of $T_{1}$ = 90 °C. The specific energy (in kJ/kg) associated with the thermal process can be calculated as $W_{th,HTST}=Q_{H,HTST}/F.$ Figure 2 presents the results of this calculation as a function of WHR efficiency. As expected, an increase in WHR reduces specific energy consumption during the pasteurization process, leading to cost savings and enhanced energy efficiency.

**Figure 3:**
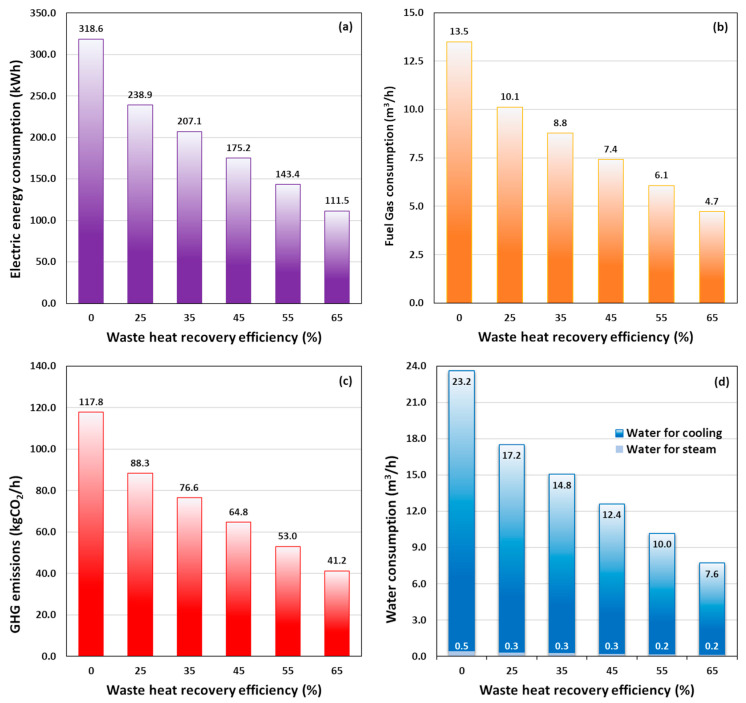
(**a**) Electrical energy consumption, (**b**) fuel gas usage, (**c**) GHG emissions, and (**d**) water consumption during the orange juice pasteurization process using HTST treatment, with varying waste heat recovery efficiency.

Figure 3a,b illustrate, respectively, the calculated consumption of electric energy and fuel gas by the steam generator as a function of WHR efficiency. As anticipated, integrating a heat recovery unit into the HTST pasteurization process for orange juice results in a substantial decrease in both electricity and gas consumption, reducing overall energy usage by more than 50% (e.g., with WHR = 55%) compared to the process without heat recovery. Figure 3c,d show the associated reductions in CO_2_ emissions and water consumption during the HTST pasteurization process, which follow a similar trend. Most of the water consumption occurs in the cooling heat exchanger, with only 2–4% of the total water volume used for steam generation. Additionally, improvements in heat recovery efficiency allow for a lower temperature $T_{3}$ of the pasteurized juice leaving the heat recovery exchanger R, which in turn reduces the volume of water needed for cooling. These reductions highlight the advancement toward more sustainable methodologies from an environmental standpoint.

#### 3.2.2. PEF Process

In the PEF pasteurization process, the juice undergoes preheating at various temperatures, denoted as $T_{1}$ (specifically 35 °C, 45 °C, and 55 °C), before entering the treatment chamber. Inside each module of the PEF chambers, the juice temperature further rises to $T_{2}$ due to the Joule effect, as illustrated in Figure 1b. In this process, the total specific energy $W_{T}$ comprises contributions from both thermal energy ($W_{th,PEF}$) and PEF energy ${(W}_{PEF,opt})$.

In particular, $W_{th,PEF}=Q_{H,PEF}/F$ values resulting from the initial preheating step (from heat exchanger *H*) at $T_{1}$ are outlined in Table S11 of the Supplementary Materials as a function of the WHR and preheating temperature. As WHR efficiency increases, the specific energy $W_{th,PEF}$ decreases. This results in a more efficient utilization of available thermal energy, leading to a reduction in the specific energy required to achieve the desired preheating temperatures $T_{1}$. For each inlet temperatures $T_{1}$, the optimal specific energy input related to the PEF treatment ($W_{PEF,opt})$, required to achieve a 5-log reduction in microbial load, are presented in Table S1 of the Supplementary Materials.

As the inlet temperature increases, the electrical energy required for juice pasteurization decreases, dropping from 102 kJ/kg at 35 °C to 38 kJ/kg at 55 °C. Table 2 shows the temperature of the juice exiting the heat recovery stage $T_{1^{\prime }}$ (refer to the schematic in Figure 1b), along with the residual thermal power $Q_{H,PEF}$ supplied to the juice in the subsequent heat exchanger (H). Here, $S_{H1}$ corresponds to the steam consumption. These values are provided as a function of the inlet temperature to the PEF chamber and WHR efficiency values, which ranges from 0 to 65%.

**Figure 4:**
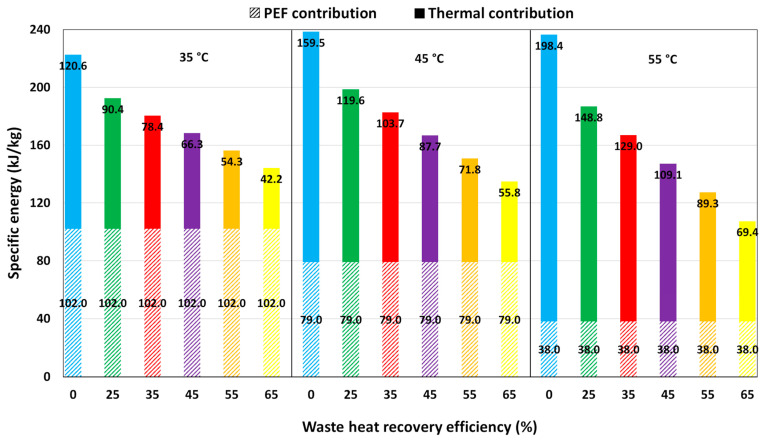
Total specific energy ${(W}_{T})$ values for the PEF pasteurization process, with varying waste heat recovery efficiency, at different preheating temperatures $T_{1}$ of the orange juice. The dashed bars correspond to the PEF contribution, whereas the homogeneously colored bars correspond to the thermal contribution.

As expected, the amount of thermal energy involved in the preheating step of juice before PEF treatment and, consequently, the utilization of fossil fuels, decrease with both increasing the inlet temperature at the same WHR and increasing WHR at constant inlet temperature. Furthermore, since PEF treatment operates at lower temperatures than the HTST process, the thermal energy consumption during heat-assisted PEF pasteurization is significantly reduced. This also promotes the “cold” pasteurization of liquid foods, which helps preserve the freshness, flavor, and nutritional properties of food products [35,55]. Moreover, this approach aligns with the growing consumer preference for minimally processed, high-quality food items [6,35,55]. Figure 4 depicts the total specific energy $W_{T}$ involved in the PEF pasteurization process, which is the sum of the thermal energy $W_{th,PEF}$ and PEF energy $W_{PEF,opt}$ contributions, as a function of heat recovery efficiency at various inlet temperatures $T_{1}$ of the orange juice.

At fixed WHR, higher $T_{1}$ increases thermal contribution while reducing the PEF energy contribution, owing to the synergistic effect of the combined heat-PEF treatment on microbial inactivation. As a result, the total specific energy $W_{T}$ decreases slightly, leading to improved process efficiency. Furthermore, as expected, at the same inlet temperature, as WHR efficiency increases, the thermal contribution decreases while the PEF contribution remains constant. This analysis provides insights into the energy dynamics of the heat-assisted PEF pasteurization process, highlighting the interplay between preheating temperature, WHR efficiency, and the respective energy contributions required for effective microbial inactivation. The use of the PEF unit for pasteurization significantly reduces the specific energy demand of the process. The resulting $W_{T}$ values are, on average, less than 20% of those recorded for thermal pasteurization treatment under various conditions (see Figure 2 for comparison).

**Figure 5:**
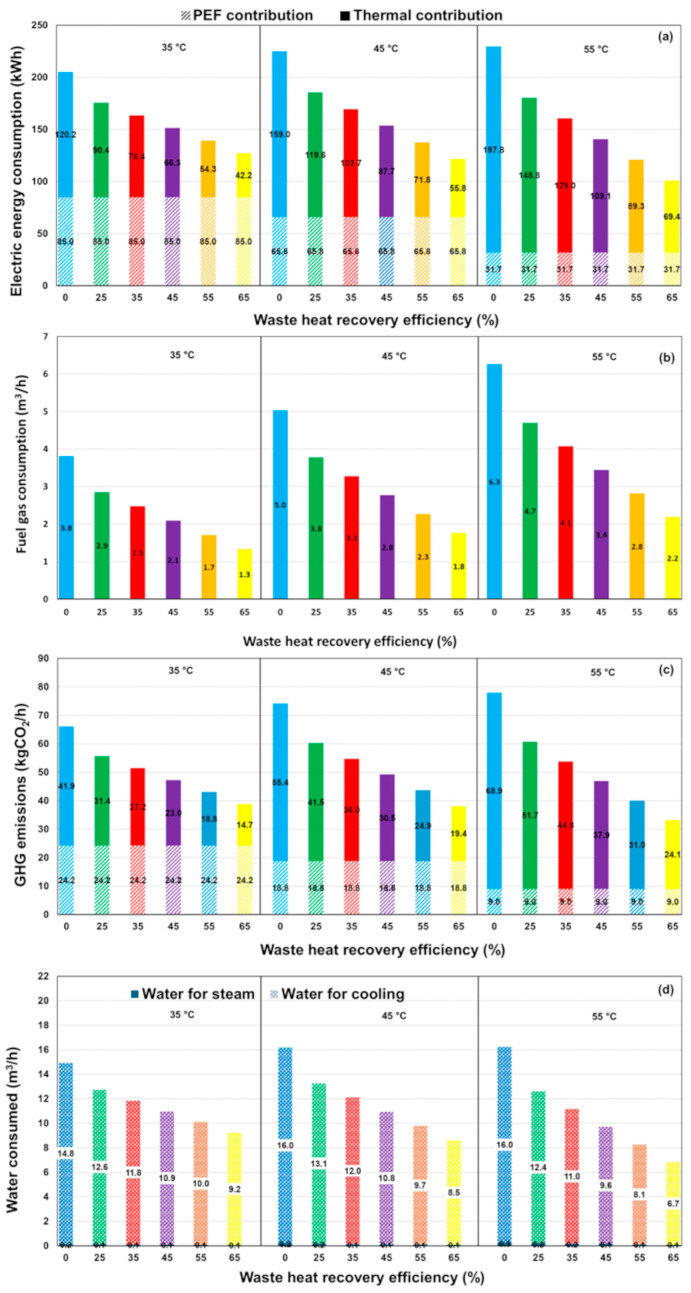
(**a**) Total electric energy consumption, (**b**) fuel gas usage, (**c**) GHG emissions, and (**d**) water consumption during the PEF pasteurization process of orange juice, with varying waste heat recovery efficiency at different preheating temperatures, $T_{1}$.

Figure 5a illustrates the total electrical energy consumption during the PEF pasteurization process as a function of heat recovery efficiencies at various inlet temperatures. This total electrical energy includes the PEF energy input ($E_{PEF}$) required to achieve the desired 5-log reduction pasteurization effect at each inlet temperature, calculated as $E_{PEF}=\left(W_{PEF,opt}\cdot F\right)/3600$. It also accounts for the thermal contribution from the electric energy consumption of the steam generator, which increases with higher inlet temperatures $T_{1}$. Notably, the overall electrical consumption for PEF pasteurization is significantly lower than that of the HTST process, as illustrated in Figure 3a for comparison. Consistent with the specific energy values, the heat-assisted PEF process reduces electrical consumption by an average of 20% compared to HTST.

Figure 5b illustrates the corresponding fuel gas consumption under the operative conditions investigated. As shown, gas consumption is entirely attributed to the steam generator, with higher preheating temperatures leading to an increase in fuel usage. Conversely, at each inlet temperature, an increase in heat recovery efficiency results in a reduction in fuel consumption. Notably, the amount of fuel gas consumed during the PEF treatment is less than 60% of that required for the HTST process (see Figure 3b for comparison).

Figure 5c illustrates the trend in GHG emissions as a function of $T_{1}$ for various heat recovery efficiencies in a PEF plant. The data reveal that, as the inlet temperature ($T_{1}$) increases, CO_2_ emissions also rise. This outcome underscores the significant impact of the preheating temperature of orange juice on the energy efficiency and associated carbon emissions of the PEF process. Higher WHR values generally lead to lower CO_2_ emissions, emphasizing the critical role of heat recovery efficiency in improving the environmental sustainability of the PEF pasteurization process. On average, emissions in the PEF process are less than 30% of those calculated for the HTST process under comparable conditions.

Figure 5d illustrates water usage during the PEF pasteurization process, by varying waste heat recovery efficiency at different preheating temperatures $T_{1}$ of the orange juice. The computed values account for the water consumption required for both steam generation and the cooling system, as described in the schematic of Figure 1b. Specifically, the volume of water used for steam generation is significantly lower than that required for the cooling system, with a maximum value of around 0.3 m^3^/h, as shown in Figure S2 of the Supplementary Materials. Here, the water usage is influenced by both WHR efficiency and the preheating temperature of the juice, $T_{1}$.

It is important to note that the juice enters the heat recovery exchanger *R* at a temperature $T_{2}=T_{1}+∆T$, where ∆T is the temperature increase of the PEF treated juice due to the Joule effect (see Table S1). As the WHR efficiency improves, more heat from the pasteurized juice is recovered, leading to a lower outlet temperature, $T_{3}$. As displayed in Figure S3 of the Supplementary Materials, the temperature of the juice entering the cooling system, $T_{3}$, decreases with higher preheating temperatures and heat recovery efficiency, eventually becoming nearly independent of the preheating temperature at higher WHR values. As a result, in the PEF process, higher WHR efficiency leads to reduced water consumption in the cooling heat exchanger C1 at all preheating temperatures. Conversely, elevating $T_{1}$ results in higher water usage. On average, the water footprint of heat-assisted PEF processing is less than 25% of that associated with the HTST thermal process due to the lower operating temperature required for PEF compared to HTST, as shown in Figure S3.

### 3.3. Cost Analysis

The economic viability of replacing commercial thermal HTST pasteurization of orange juice at 90 °C for 15 s with a heat-assisted PEF process has been assessed. A comprehensive cost model has been developed and applied across various WHR efficiency values ranging from 0 to 65% for both processes and three different inlet temperatures to the PEF chambers (35 °C, 45 °C, and 55 °C). Table 3 provides an example of the adopted framework, encompassing initial investment costs $C_{0}$, alongside expenses related to utility, labour, and facilities for the analyzed pasteurization processes. It highlights the operating conditions under which both technological solutions achieve a thermal recovery rate of 35%, with a processing temperature $T_{1}$ set to 90 °C for the HTST and 55 °C for PEF. While similar modeling approaches to assess the cost analysis of a production plant are reported in the literature, few and outdated studies specifically address a comparative cost analysis of HTST and PEF commercial pasteurization processes for liquid foods [24,31,32,44,56,57,58].

The capital costs have been estimated based on equipment prices provided by an equipment manufacturing company: EUR 680,000 for the PEF system and EUR 200,000 for the HTST system. Additional costs have been calculated at EUR 70,666 for the PEF system and EUR 90,555 for the HTST system covering pumps, heat exchangers for juice cooling, and steam generators. Furthermore, the estimation includes expenses for equipment installation, commissioning, provision of necessary piping, electrical components, and process control systems. The utility costs (electrical energy, natural gas, and water) for pasteurizing orange juice with each technology have been calculated based on thermal recovery efficiency and temperature treatment, as outlined in the schematics depicted in Figure 1. These costs have been determined using the following unit rates: EUR 3.5/m^3^ for water, EUR 0.6/smc for natural gas, and EUR 0.25/kWh for electricity.

**Figure 6:**
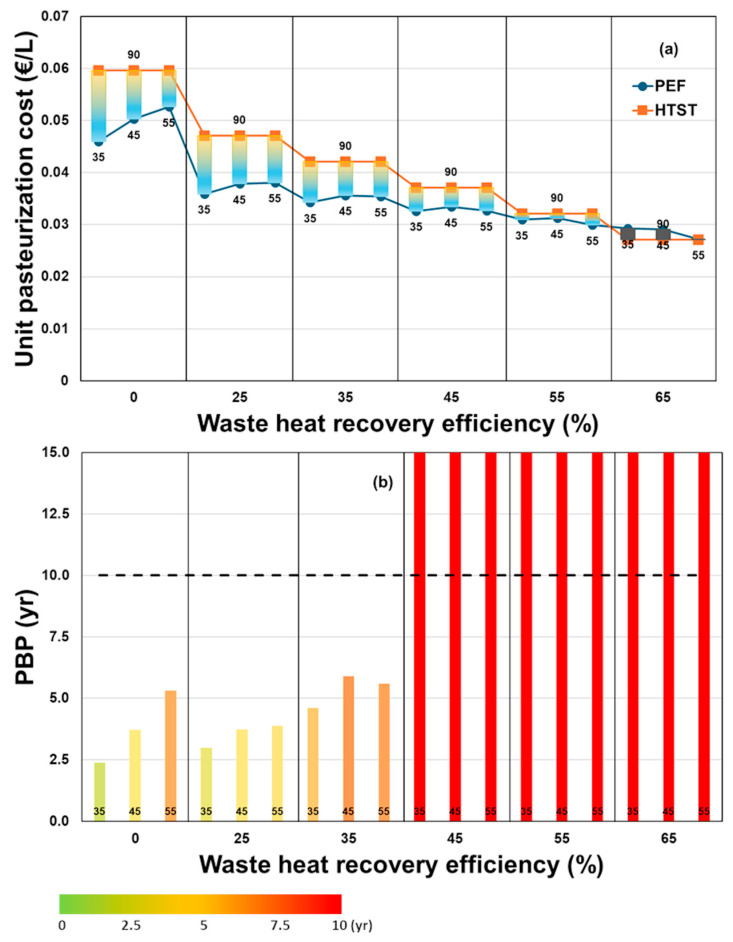
(**a**) A comparison of the unit pasteurization cost of orange juice using both PEF and HTST processing, and (**b**) the trend of the payback period (PBP) as a function of waste heat recovery (WHR) efficiency and preheating temperature for the PEF process. The temperatures of the processes are reported as labels close to the indicators. The color scale indicates different payback periods: dark green for PBP from 0 to 2.5 years, light green for PBP from 2.5 to 5 years, orange for PBP from 5 to 7.5 years, and red for PBP greater than 10 years.

As illustrated in Table 3, equipment capital costs are the primary factor driving overall production expenses. Notably, the commercial PEF process achieves a lower unit pasteurization cost (3.5 cents/L) compared to the reference thermal process (4.2 cents/L), aligning with values reported in the literature [24]. Additionally, the heat-assisted PEF process results in annual savings of approximately EUR 110,000 compared to thermal treatment. The associated NPV, calculated using Equation (4), is positive, as presented in Table S12, indicating that implementing the PEF process could yield substantial annual cost savings over the reference thermal process. Figure 6a illustrates the variation in unit pasteurization costs for both technologies across different WHR efficiencies and treatment temperatures. The data reveal that, under all evaluated operating conditions except for the case with WHR of 65%, the pasteurization unit cost using heat-assisted PEF technology is lower than that of conventional thermal treatment. As WHR efficiency increases, the operational costs of thermal pasteurization decrease significantly, narrowing the cost gap between the two technologies. Similarly, raising the preheating temperature (*T*_1_) in the PEF process reduces its cost advantage, primarily due to higher utility expenses. However, this trend is observed only when WHR efficiency remains below 45%. At higher WHR levels, operating at the maximum preheating temperature of 55 °C leads to a slight reduction in unit pasteurization costs for the PEF process. This is likely due to enhanced microbial inactivation efficiency and improved heat recovery performance at elevated temperatures. Moreover, a comprehensive analysis should account for the added value of PEF-treated products compared to HTST, particularly regarding potential quality enhancements such as improved nutrient retention and sensory attributes. These factors could significantly boost the marketability and consumer appeal of PEF over conventional thermal treatments.

Using Equation (5), the payback period (PBP) for replacing the thermal pasteurization of orange juice with a heat-assisted PEF solution has been calculated. Figure 6b illustrates the variation in PBP as a function of thermal recovery efficiency at different preheating temperatures $T_{1}$, factoring in the unit costs of water, natural gas, and electricity.

This analysis underscores the significant impact of WHR and preheating conditions on the economic viability of the PEF process. The overall results indicate that, at fixed WHR levels, increasing the preheating temperature generally leads to a longer payback period (PBP), highlighting the reduced cost advantage of the PEF solution and the increasing economic competitiveness of HTST. However, the impact of preheating temperature is strongly dependent on the WHR level. At low WHR values (<25%), the PBP consistently increases with rising preheating temperatures, ranging from 2.4 to 5.3 years at 0% WHR and from 3.0 to 3.9 years at 25% WHR as the temperature increases from 35 °C to 55 °C. At 35% WHR, a non-monotonic trend is observed: the PBP rises from 4.6 to 5.9 years as the preheating temperature increases from 35 °C to 45 °C, but then slightly decreases to 5.6 years at 55 °C. This reduction aligns with the slight decrease in the unit pasteurization cost of PEF shown in Figure 6a and may be attributed to improved microbial inactivation efficiency and increased heat recovery at higher temperatures. At WHR levels of 45% and above, the investment in PEF technology becomes economically unfeasible within a reasonable timeframe, as the PBP exceeds 10 years (NPV < 0). These conditions reflect significantly reduced economic viability, where cost recovery would require an extended period, classifying the investment as non-beneficial.

### 3.4. Energy Cost Sensitivity Analysis

To explore various energy scenarios, the study examines fluctuations in electricity and natural gas costs, keeping water costs constant. This sensitivity analysis quantifies the impact of energy price variations on key economic indicators (NPV and PBP) of the proposed PEF system. Details of these scenarios are provided in Table S13 of the Supplementary Materials. Specifically, Scenario 1 represents lower energy (EUR 0.25/kWh) and natural gas (EUR 0.6/smc) costs, whereas Scenario 4 reflects higher costs. Scenarios 2 and 3 focus on the individual impacts of energy and gas cost variations.

**Figure 7:**
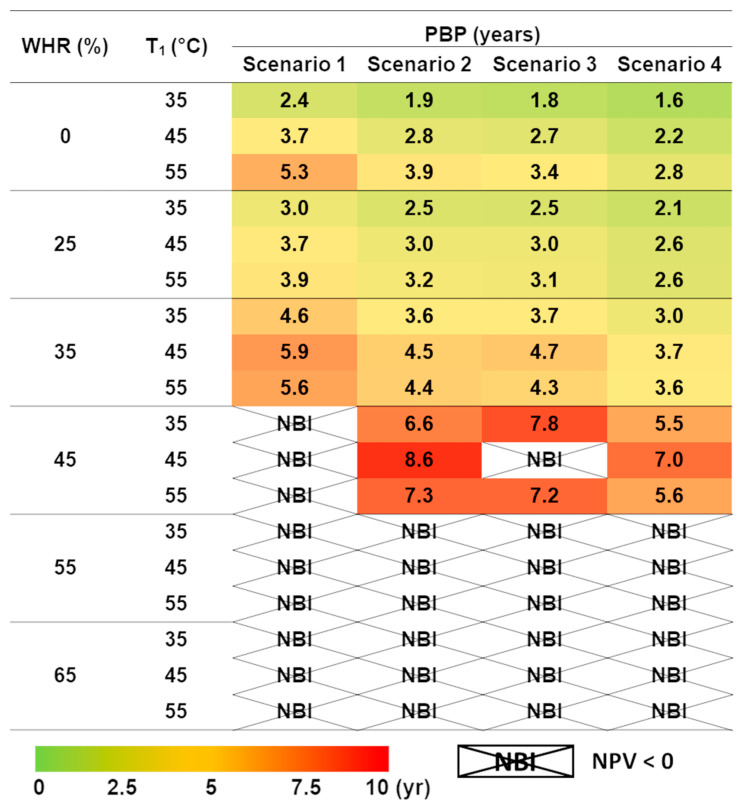
Sensitivity analysis: Payback periods calculated for various energy cost scenarios resulting from the implementation of PEF technology in the pasteurization process of orange juice, replacing the conventional HTST thermal treatment. Different scenarios reflect fluctuations in electricity and natural gas prices to assess their impact on economic feasibility. The color scale indicates different payback periods: light green for PBP from 0 to 2.5 years, dark green for PBP from 2.5 to 5 years, orange for PBP from 5 to 7.5 years, and red for PBP greater than 10 years. NBI stands for Not Beneficial Investment, with a negative NPV computed from Equation (4).

Figure 7 shows the computed payback periods for various energy cost scenarios during the transition from conventional HTST to a PEF-based system for orange juice processing. The preheating temperature and WHR efficiency have been selected to ensure a maximum PBP of 10 years alongside a positive NPV.

A negative NPV, calculated using Equation (4), is denoted as a “Not Beneficial Investment” (NBI). According to the results shown in Figure 6, Scenario 1 represents the least advantageous case, resulting in a negative NPV for WHR levels above 35%, regardless of the preheating temperature. In contrast, increases in electricity and natural gas prices, as reflected in Scenarios 2 through 4, lead to more attractive PBP outcomes, achieving acceptable values even at a WHR of 45%. Notably, Scenario 4, which assumes the highest energy costs, consistently shows shorter PBP values compared to other scenarios under similar conditions (same preheating temperature and WHR), highlighting improved economic viability in high energy cost contexts. These findings confirm the economic robustness of the PEF-based pasteurization process against energy price fluctuations. Despite variations across Scenarios 1 to 4, the relative advantage compared to conventional HTST remains stable, reinforcing the reliability of the economic assessment under different market conditions.

**Figure 8:**
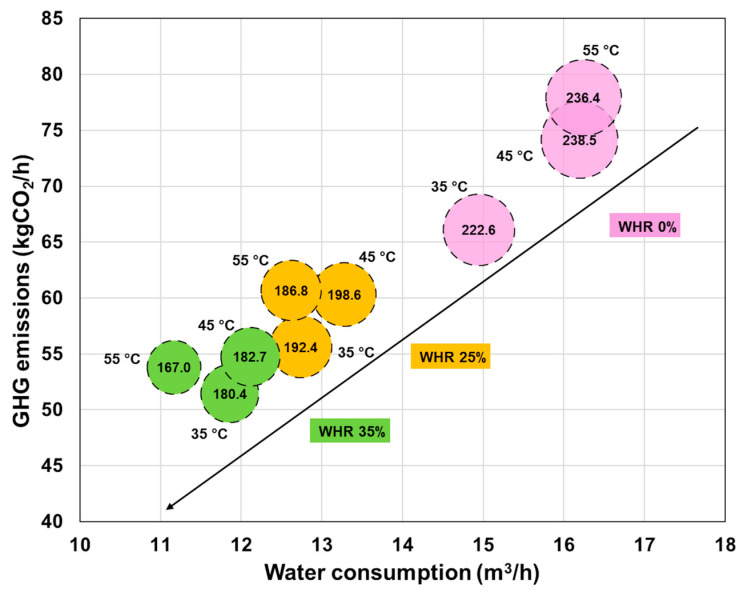
Correlation between GHG emissions and water footprint at various preheating temperatures and thermal recovery efficiency levels for the PEF plant. The data are segmented into three datasets, each associated with distinct total specific energy values $W_{T}$, indicated as labels within the bubbles. The arrow shows the increase in WHR values. The data shown are selected based on Scenario 1.

Among the configurations analyzed in Figure 7, the most environmentally friendly configuration for a PEF plant to replace the conventional HTST pasteurization can be selected by evaluating sustainable factors such as CO_2_ emissions and water consumption. By analyzing the correlation between GHG emissions and water usage, a comparative visualization has been developed to demonstrate how various configurations impact environmental sustainability and resource efficiency. This analysis highlights critical trade-offs, guiding the selection of a PEF plant that achieves an optimal balance between economic performance and environmental impact. Figure 8 depicts the relationship between GHG emissions and water footprint across different preheating temperature T_1_ and WHR efficiency values for the PEF plant. The data are grouped into three distinct categories, each representing a particular WHR efficiency level having a short payback period (PBP ≤ 6 years) in Scenario 1. The optimal PEF plant configuration minimizes both water consumption and CO_2_ emissions while balancing sustainability and resource efficiency.

As shown in Figure 8, a PEF setup with a WHR of 35% and a preheating temperature of 55 °C emerges as the most energy-efficient and sustainable configuration among the evaluated scenarios. This condition yields the lowest specific energy consumption (167.0 kJ/kg) and water usage (11.2 m^3^/h), while also minimizing CO_2_ emissions (53.8 kg/h). Compared to lower preheating temperatures at the same WHR level, the 55 °C setting offers clear advantages in terms of overall resource efficiency. Therefore, a 35% WHR combined with a preheating temperature of 55 °C has been identified as the optimal operating point in this case study, offering the best balance between energy consumption, microbial inactivation, and overall sustainability.

However, future access to more robust and reliable inactivation data, validated at an industrial scale rather than relying on the currently available literature data used in this study, could further refine our findings. For instance, evaluating microbial inactivation using a diverse cocktail of microorganisms, including bacteria (e.g., *E. coli*, *Salmonella* spp., *L. monocytogenes*), yeasts (e.g., *S. cerevisiae*), and molds (e.g., *Penicillium* spp., *Aspergillus* spp.) typically found in fruit juice [15,16,36,59], rather than relying on a single strain, would account for variability among microorganisms, ensuring broader efficacy and reliability [36]. Additionally, it is important to note that inactivating inoculated microorganisms is generally easier than eliminating endogenous microflora in fruit juice [60,61]. This difference is primarily due to the adaptation of native microbes to the juice environment, which can enhance their resistance to stressors such as acidity, antimicrobial compounds, and processing conditions. In such cases, the lower sensitivity of native microflora in fruit juice to PEF treatment, compared to inoculated microorganisms, could be offset by the enhanced synergistic effect of electric pulses and temperatures above 35 °C on microbial inactivation, as supported by literature findings [16,36,62].

### 3.5. Environmental Impacts of Orange Juice Pasteurization Methods

#### 3.5.1. Resource Usage

The life cycle inventory (LCI) analysis summarized in Table S10 has been performed for the optimal configuration, with a preheating temperature of 55 °C and a recovery efficiency of 35%. The LCI highlights notable differences in resource consumption between conventional pasteurization and heat-assisted PEF pasteurization for orange juice production. Specifically, as shown in Table S10, conventional pasteurization consumes 0.069 kWh of electricity, 0.003 m^3^ of natural gas, and 0.005 m^3^ of water per liter of orange juice, whereas the heat-assisted PEF method requires only 0.054 kWh of electricity, 0.001 m^3^ of natural gas, and 0.004 m^3^ of water per liter of orange juice.

These results highlight significant reductions in resource use with the heat-assisted PEF process: electricity consumption is reduced by 22.5%, natural gas usage decreases by 53.8%, and water consumption drops by 25.2%. Such reductions underscore the potential of innovative pasteurization technologies to improve resource efficiency, promoting more sustainable production practices in the food industry.

#### 3.5.2. Midpoint Environmental Impact Indicators

**Figure 9:**
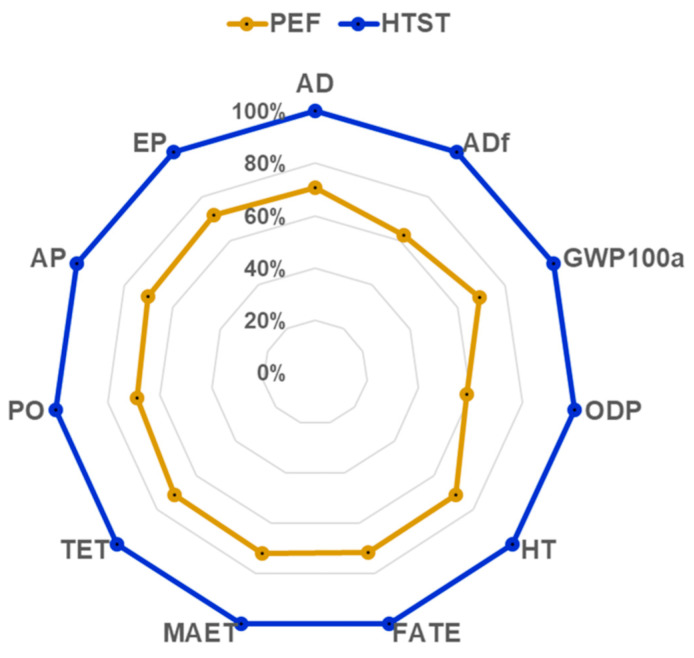
Environmental impact reduction across various categories achieved through the application of heat-assisted pulsed electric field pasteurization compared to conventional high-temperature short-time pasteurization for the pasteurization of one liter of orange juice.

The environmental impacts of producing 1 L of pasteurized orange juice using conventional HTST pasteurization and heat-assisted PEF pasteurization methods have been evaluated through several midpoint indicators. Table 4 summarizes the results of this evaluation, offering insights into resource consumption and emissions for each method across various impact categories. Additionally, Figure 9 illustrates the reduction in environmental impacts achieved with novel pasteurization technologies.

The results consistently demonstrate that PEF pasteurization outperforms HTST in reducing environmental impacts, with reductions ranging from 28% to 42% across different categories. The application of PEF technology significantly lowers resource consumption and associated emissions. The most critical categories impacted by this technology have been detailed further.

Global warming potential (GWP) measures the contribution of the process to climate change by quantifying its greenhouse gas emissions over a 100-year period (GWP100a). In this study, conventional HTST pasteurization generates 0.0312 kg CO_2_ eq per liter of orange juice, while the heat-assisted PEF method results in only 0.0216 kg CO_2_ eq, reflecting a 31% reduction in GWP with PEF technology. The environmental assessment also reveals significant reductions in fossil fuel consumption (EDf). Conventional HTST pasteurization consumes 0.483 MJ of fossil fuel energy per liter, whereas the heat-assisted PEF method requires just 0.302 MJ, resulting in a 37% reduction in fossil fuel depletion.

PEF technology also contributes to a decrease in ozone depletion potential (ODP). The ODP for HTST pasteurization is 5.54 × 10^−9^ kg CFC-11 eq, while the heat-assisted PEF method reduces this to 3.23 × 10^−9^ kg CFC-11 eq, resulting in a 42% decrease in ozone depletion potential. Human toxicity (HT), which evaluates the potential harm caused by chemicals to human health, shows a 29% reduction with PEF technology. HTST generates 6.54 × 10^−3^ kg 1,4-DB eq, while the heat-assisted PEF method reduces this to 4.66 × 10^−3^ kg 1,4-DB eq, highlighting the potential of PEF to mitigate harmful chemical exposure. Acidification potential (AP), caused by the emission of pollutants such as sulfur dioxide (SO_2_), nitrogen oxides (NO_x_), and ammonia (NH_3_), is also significantly reduced by PEF technology. HTST has an AP of 1.64 × 10^−4^ kg SO_2_ eq, whereas the heat-assisted PEF method reduces this to 1.15 × 10^−4^ kg SO_2_ eq, representing a 30% reduction in acidification potential. Eutrophication potential (EP), which refers to nutrient enrichment in water bodies that can lead to harmful algal blooms and oxygen depletion, also sees a significant reduction. In this study, EP decreases by 29%, from 4.67 × 10^−5^ kg PO_4_^3−^ eq for HTST to 3.34 × 10^−5^ kg PO_4_^3−^ eq for the heat-assisted PEF method, demonstrating the effectiveness of PEF in minimizing water pollution impacts.

Thermal units in the food industry are significant contributors to environmental impacts due to their high consumption of methane and electricity, which contribute to air pollution, smog, and acid rain. The combustion of fossil fuels in these units also disrupts ecosystems and pollutes air and water. Additionally, electricity production, largely dependent on fossil fuels, is a major contributor to climate change. The adoption of innovative technologies such as PEF, which reduce fossil fuel and electricity usage, can significantly mitigate these environmental impacts.

This study underscores the potential of heat-assisted PEF technology to offer substantial environmental benefits over conventional pasteurization. Its application not only enhances resource efficiency but also supports more sustainable food processing practices, marking it as a promising alternative in the production of pasteurized orange juice.

### 3.6. Regulatory Challenges in the Adoption of PEF Technology

The adoption of PEF technology in food processing is subject to a range of regulatory challenges that differ significantly across geographic regions due to varying food safety standards, risk assessment criteria, and legal frameworks. These challenges are critical for manufacturers to address to effectively implement PEF technology in commercial production systems [63,64,65,66,67,68]. In the United States, the FDA regulates food processing technologies, including PEF. Products treated with PEF may require Generally Recognized as Safe (GRAS) status or a food additive petition, both of which necessitate the submission of scientific evidence demonstrating safety. GRAS status involves consensus among qualified experts and can represent a time-consuming and complex pathway [64,68].

In the European Union, the European Food Safety Authority (EFSA) and the European Commission regulate novel food technologies under Regulation (EU) 2015/2283. PEF-treated foods may be classified as novel foods if the process leads to significant changes in structure or composition, requiring pre-market authorization and comprehensive safety, nutrition, and consumer perception assessment [68]. At the international level, while the Codex Alimentarius does not yet specifically classify PEF technology, its general principles of food hygiene provide a foundation for assessing the safety of new processing techniques. Although Codex guidelines are not legally binding, they influence national regulatory policies and can facilitate international market access [69].

Regulatory frameworks vary in Canada, Australia and New Zealand, and many Asian countries. For example, Health Canada may require pre-market notification if PEF alters the properties of the food, Food Standards Australia New Zealand (FSANZ) evaluates such technologies on a case-by-case basis [70], and Asian regulatory authorities often lack well-defined pathways for PEF, resulting in greater uncertainty [71]. A comparative summary of these regional regulatory approaches is provided in Table S14 of the Supplementary Materials.

In addition to regulatory classification and approval procedures, labeling requirements also affect the adoption of PEF technology. Currently, there are no specific labeling mandates for PEF-treated foods in key markets such as the U.S. and EU. This regulatory gap may contribute to consumer uncertainty, as a lack of transparency can hinder acceptance of new technologies [64,68]. Developing informative labeling strategies could help mitigate public skepticism and increase consumer trust. Furthermore, all regulatory bodies require evidence that PEF processing does not compromise food safety, nutritional quality, or sensory characteristics. This involves extensive testing, including microbial validation and chemical stability studies, which can pose a significant burden on food producers and delay market entry [27,72].

PEF technology holds promise for food preservation, but its adoption is limited by fragmented regulations. Progress depends on early regulatory engagement, solid scientific evidence, and transparent consumer communication.

## 4. Conclusions

This study evaluated the feasibility of integrating pulsed electric field technology with heat recovery for fruit juice pasteurization, considering energy, economic, and sustainability factors. On average, compared to conventional HTST pasteurization (90 °C for 15 s), the PEF with moderate preheating (35–55 °C) and partial heat recovery reduced electricity and fuel consumption by 20% and 60%, respectively, leading to ~30% lower GHG emissions and a 25% reduction in water consumption. A sensitivity analysis identified optimal PEF configurations to replace thermal pasteurization, highlighting that 35% WHR offered the best energy efficiency and sustainability. Despite higher initial investment costs, improved efficiency shortened the payback period (≤6 years) and minimized environmental impact. The optimal configuration, PEF at 55 °C, provided the best balance of microbial inactivation and sustainability. Life cycle assessment showed reductions of 28–42% across impact categories, with global warming potential (GWP) decreasing by 31% and fossil fuel depletion by 37%.

Overall, the findings of this study underscore the potential of PEF as a sustainable alternative for food processing. To support its commercial implementation, further research, particularly in collaboration with industry partners, is needed to refine LCA data, expand the analysis to additional case studies, and strengthen the basis for informed decision-making. In addition, addressing scale-up challenges is crucial, highlighting the need for further technological advancements to increase the processing capacity of PEF systems for preservation purposes.

## Figures and Tables

**Table 1:** Preheating temperature $T_{1^{\prime }}$, residual thermal power $Q_{H,HTST}$, and steam consumption during the HTST pasteurization process of orange juice at $T_{1}$ = 90 °C as a function of the waste heat recovery (WHR) efficiency.

Waste Heat Recovery (WHR) Efficiency (%)	$T_{1\prime }$ (°C)	$Q_{H,HTST}$* (kW)	$S_{H1}$** (kg/h)
0	4.0	278.8	456.0
25	25.5	209.1	342.0
35	34.1	181.2	296.4
45	42.7	153.3	250.8
55	51.3	125.5	205.2
65	59.9	97.6	159.6

* $Q_{H,HTST}=F\cdot C_{ps}\cdot \left(T_{1}-T_{1\prime }\right)$, where *F* is the flow rate and $C_{ps}$ is the specific heat capacity of orange juice, assumed to be 3.89 kJ/kg °C [35]. **${{\;S}_{H}=Q}_{H,HTST}/\lambda_{H1},$ where $\lambda_{H1}$ = 2201 kJ/kg represents the latent heat of steam at 120 °C at the operating pressure of 2 bar [1].

**Table 2:** Preheating temperature $T_{1^{\prime }}$ residual thermal power $Q_{H,PEF}$, and steam consumption during the PEF pasteurization process of orange juice at different inlet temperature ${(T}_{1})$ to the PEF chamber as a function of the waste heat recovery (WHR) efficiency. Here, ${S_{H}=Q}_{H,PEF}/\lambda_{H1},$ where $\lambda_{H1}$ = 2257 kJ/kg represents the latent heat of steam at 100 °C within the operating pressure of 1 bar [1].

Waste Heat Recovery (WHR) Efficiency (%)	$T_{1}$ (°C)	$T_{1\prime }$ (°C)	$Q_{H,PEF}$ (kW)	$S_{H1}$ (kg/h)
0	35	4.0	100.5	160.3
	45	4.0	132.9	212.0
	55	4.0	165.3	263.7
25	35	11.8	75.4	120.2
	45	14.3	99.7	159.0
	55	16.8	124.0	197.8
35	35	14.9	65.3	104.2
	45	18.4	86.4	137.8
	55	21.9	107.5	171.4
45	35	18.0	55.3	88.2
	45	22.5	73.1	116.6
	55	27.0	90.9	145.0
55	35	21.1	45.2	72.1
	45	26.6	59.8	95.4
	55	32.1	74.4	118.7
65	35	24.2	35.2	56.1
	45	30.7	46.5	74.2
	55	37.2	57.9	92.3

**Table 3:** Costs of the commercial PEF and thermal HTST pasteurization process for orange juice.

Process Parameters	HTST $(\mathrm{WHR}\;35\%,\;T_{1}$ = 90 °C)	PEF (WHR 35%, $T_{1}$ = 55 °C)
Process flow (L/yr)	16,500,000	16,500,000
Annual hours of production (h/yr)	5500	5500
Throughput (L/h)	3000	3000
	**Capital costs**	
Heat exchangers (EUR)	4500	5500
PEF equipment (EUR)	0	680,000
HTST equipment (EUR)	200,000	0
Process chilling (EUR)	28,830	35,340
Process pumps (EUR)	1000	1000
Gas steam generator (EUR)	56,525	28,826
**Total capital cost (EUR)**	**290,855**	**750,666**
	**Utility costs**	
Process electricity (kWh/yr)	1,138,926	881,211
Steam natural gas (smc/yr)	48,252	22,390
Cooling water (m^3^/yr)	58,021	29,139
Water for steam (m^3^/yr)	1630	943
Electric charges (EUR/yr)	284,731	220,303
Steam natural gas (EUR/yr)	28,951	13,434
Cooling water charges (EUR/yr)	203,073	101,986
Water for steam charges (EUR/yr)	5705	3300
**Annual utility costs (EUR/yr)**	**522,462**	**339,022**
	**Labour costs**	
Plant operators per shift	1	1
Labor costs (EUR/h)	23.25	23.25
**Annual labour costs (EUR/yr)**	**127,875**	**127,875**
	**Facility-related costs**	
Estimated plant life (years)	10	10
Maintenance charges (%)	2	3
Administration charges (%)	2.5	2.5
Annual depreciation ^§^ (EUR/yr)	29,086	75,067
Maintenance and administration charges (EUR/yr)	13,088	41,287
**Facility related costs (EUR/yr)**	**42,174**	**116,353**
**Total annual costs (EUR/yr)**	**692,511**	**583,250**
**Unit pasteurization cost (EUR/L)**	**0.042**	**0.035**

^§^ Here, the annual depreciation rate is set to 10%.

**Table 4:** Environmental impact of conventional HTST and heat-assisted PEF for pasteurization of one liter of orange juice.

Impact Category	Acronym	Unit	HTST	PEF
**Abiotic Depletion**	AD	kg Sb eq	1.02 × 10^−8^	7.22 × 10^−9^
**Abiotic Depletion (fossil fuels)**	ADf	MJ	4.83 × 10^−1^	3.02 × 10^−1^
**Global Warming Potential**	GWP100a	kg CO_2-eq_	3.12 × 10^−2^	2.16 × 10^−2^
**Ozone Layer Depletion**	ODP	kg CFC-11 eq	5.54 × 10^−9^	3.23 × 10^−9^
**Human Toxicity**	HT	kg 1,4-DB eq	6.54 × 10^−3^	4.66 × 10^−3^
**Fresh Water Aquatic Ecotox.**	FAET	kg 1,4-DB eq	6.42 × 10^−3^	4.60 × 10^−3^
**Marine Aquatic Ecotoxicity**	MAET	kg 1,4-DB eq	2.51 × 10^1^	1.80 × 10^1^
**Terrestrial Ecotoxicity**	TET	kg 1,4-DB eq	3.73 × 10^−5^	2.65 × 10^−5^
**Photochemical Oxidation**	PO	kg C_2_H_4_ eq	6.61 × 10^−6^	4.54 × 10^−6^
**Acidification**	AP	kg SO_2_ eq	1.64 × 10^−4^	1.15 × 10^−4^
**Eutrophication**	EP	kg PO_4_^3−^ eq	4.67 × 10^−5^	3.34 × 10^−5^

## Data Availability

The original contributions presented in the study are included in the article/Supplementary Material, further inquiries can be directed to the corresponding authors.

## Supplementary Materials

The following supporting information can be downloaded at: https://www.mdpi.com/article/10.3390/foods14132239/s1, Table S1: Optimal specific electrical energy $W_{PEF,opt}$ related to the PEF pasteurization process of orange juice at various inlet temperatures $T_{1}$ required to achieve a 5-log reduction in microbial load [36]. ∆T is the corresponding temperature increase of the juice during PEF treatment due to the Joule effect calculated using Equation (2); Figure S1: Simplified schematic of a heat exchanger; Table S2: Key equations and thermal parameters applied in the heat transfer analysis and design of each heat exchanger depicted in the schematic of Figure 1; Table S3. Heat transfer and design calculations for the heat recovery exchanger (R) used in the conventional thermal HTST pasteurization process of orange juice (Figure 1a), as a function of the waste heat recovery (WHR) efficiency; Table S4: Heat transfer and design calculations for the heat exchanger (H) used in the conventional thermal HTST pasteurization process of orange juice (Figure 1a), as a function of the waste heat recovery (WHR) efficiency; Table S5: Heat transfer and design calculations for the cooling heat exchanger (C1) used in the conventional thermal HTST pasteurization process of orange juice (Figure 1a), as a function of the waste heat recovery (WHR) efficiency; Table S6: Heat transfer and design calculations for the heat recovery exchanger (R) used in the PEF system for pasteurization of orange juice (Figure 1a), as a function of the waste heat recovery (WHR) efficiency; Table S7: Heat transfer and design calculations for the heat exchanger (H) used in the PEF system for pasteurization of orange juice, as illustrated in the schematic of Figure 1a, as a function of the waste heat recovery (WHR) efficiency; Table S8: Heat transfer and design calculations for the cooling heat exchanger (C2) used in the PEF system for pasteurization of orange juice, as illustrated in the schematic of Figure 1a, as a function of the waste heat recovery (WHR) efficiency; Table S9: Heat transfer and design calculations for the cooling heat exchanger (C1) used in the PEF system for pasteurization of orange juice, as illustrated in the schematic of Figure 1a, as a function of the waste heat recovery (WHR) efficiency; Table S10: Life cycle inventory analysis of resource consumption for pasteurizing one liter of orange juice by HTST and heat-assisted PEF process with a preheating temperature of 45 °C and a recovery efficiency of 35%; Table S11: Specific thermal energy ($W_{th})$ values for the PEF pasteurization process supplied during preheating of orange juice at different inlet temperatures ${(T}_{1})$ to the PEF chambers as a function of the waste heat recovery (WHR) efficiency; Figure S2: Water consumption for the steam generation during the PEF pasteurization process as a function of the waste heat recovery efficiency at different preheating temperatures $T_{1}$ of the orange juice; Figure S3: Temperature profiles of PEF-pasteurized orange juice during the thermal recovery step, with varying preheating temperature $T_{1}$ and waste heat recovery (WHR) efficiencies; Table S12: Computed NPV values for the PEF pasteurization process at different combinations of preheating temperatures $T_{1}$ and waste heat recovery (WHR) efficiency; Table S13: Energy scenarios based on electricity and natural gas costs, with water costs fixed at EUR 3.5/m^3^; Table S14: Comparative regulatory frameworks for PEF-treated juices.
