# Supplementary material for: Comparative Analysis of Cost, Energy Efficiency, and Environmental Impact of Pulsed Electric Fields and Conventional Thermal Treatment with Integrated Heat Recovery for Fruit Juice Pasteurization
Source: Foods. 2025 Jun 25;14(13):2239. doi: 10.3390/foods14132239 (PMC12248458; doi:10.3390/foods14132239)
Supplement: Supplementary file 1 [file foods-14-02239-s001.zip › foods-3621915-supplementary.pdf]

foods

Article

Comparative Analysis of Cost, Energy Efficiency and Environmental Impact of Pulsed Electric Fields and Conventional Thermal Treatment with Integrated Heat Recovery for Fruit Juice Pasteurization

Giovanni Landi [1,*], Miriam Benedetti [2], Matteo Sforzini [2], Elham Eslami [3], and Gianpiero Pataro [3,*]

[1] ENEA, Portici Research Center, Piazzale Enrico Fermi, Località Granatello, 80055 Portici, Italy;
[2] ENEA, Casaccia Research Center, Via Anguillarese 301, 00123 Rome, Italy;
[3] Department of Industrial Engineering, University of Salerno, Via Giovanni Paolo II, 132, 84084, Fisciano, SA, Italy;

* Correspondence: giovanni.landi@enea.it (G.L.); gpataro@unisa.it (G.P.)

1. Optimal PEF energy input and temperature increase

Table S1. Optimal specific electrical energy $W_{PEF,opt}$ related to the PEF pasteurization process of orange juice at various inlet temperatures T_1 required to achieve a 5-log reduction in microbial load [20]. ΔT is the corresponding temperature increase of the juice during PEF treatment due to the Joule effect calculated using Equation (2).

T_1 (°C)	$W_{PEF,opt}$ (kJ/kg)	ΔT (°C)
35	102	12.5
45	79	9.6
55	38	4.6

Academic Editor: Firstname Lastname

Received: date

Revised: date

Accepted: date

Published: date

Citation: To be added by editorial staff during production

Copyright: © 2025 by the authors. Submitted for possible open access publication under the terms and conditions of the Creative Commons Attribution (CC BY) license (https://creativecommons.org/licenses/by/4.0/).

2. Heat Transfer Analysis and Design Calculations for Heat Exchangers

Figure S1 presents a general schematic of each heat exchanger included in Figure 1, indicating the flow rates and temperatures of both the hot (h) and cold (c) streams.

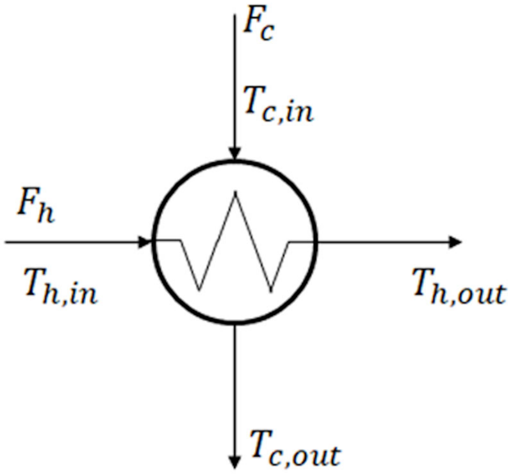

Figure S1. Simplified schematic of a heat exchanger.

Table S2 summarizes the main equations and thermal parameters used for heat transfer and design calculations for each heat exchanger included in the schematic shown in Figure 1

Table S2. Key equations and thermal parameters applied in the heat transfer analysis and design of each heat exchanger depicted in the schematic of Figure 1.

Equations/Thermal parameters	Descriptions
$Q_h = F_h \cdot C_{p,h} \cdot (T_{h,in} - T_{h,out})$	Thermal power based on the heat balance of the hot stream (in W)
$Q_c = F_c \cdot C_{p,c} \cdot (T_{c,out} - T_{c,in})$	Thermal power based on the heat balance of the cold stream (in W)
$Q = Q_h = Q_c = U \cdot A \cdot LMTD$	Heat Transfer Rate Equation for Heat Exchangers (in W)
$LMTD = \frac{(T_{h,in} - T_{c,out}) - (T_{h,out} - T_{c,in})}{LN\left(\frac{T_{h,in} - T_{c,out}}{T_{h,out} - T_{c,in}}\right)}$	Logarithmic mean temperature difference (in °C)
$S_H = Q_H/\lambda_H,$	Steam consumption (in kh/h) during heating of orange juice in the heat exchanger H (Figure 1)
λ_H= 2201 kJ/kg	Latent heat of saturated steam at 120°C (used for HTST process)

λ_H= 2257 kJ/kg	Latent heat of saturated steam at 100°C (used for PEF process)
$C_{p,h}$ = 3.89 kJ/kg °C (*)	Specific heat capacity of orange juice
$C_{p,c}$ = 4.18 kJ/kg °C.	Specific heat capacity of water

(*)[1,32]

Hypothesis and optimization criteria

1. Plate heat exchanger operating in counterflow were considered.
2. The energy losses in the thermal balance of each heat exchanger in both PEF and HTST processes were not considered in this study.
3. Where applicable, process parameters were selected to achieve log mean temperature difference (LMTD) values ranging from 10°C to 30°C, ensuring efficient heat exchanger performance [1,32].
4. For efficient chiller operation, supplying cold water at 2°C to heat exchangers C1 and C2 (Figure 1), and to maintain good thermal efficiency (LMTD) in both units, the cold-water outlet temperature (Tc2), returning to the chiller, was set at 12°C [37].
5. The cost of each heat exchanger shown in Figure 1 has been estimated based on the type of fluids involved, the exchanged thermal power, and an exchange area falling within the range calculated in Tables S3–S9.

Calculation for heat exchanger of involved in the HTST pasteurization process of orange juice (Figure 1a)

Table S3. Heat transfer and design calculations for the heat recovery exchanger (R) used in the conventional thermal HTST pasteurization process of orange juice (Figure 1a), as a function of the waste heat recovery efficiency WHR.

WHR (%)	T_o (°C)	$T_{1'}$ (°C)	T_1 (°C)	T_2 (°C)	$Q_{R,HTST}$ (kW)	T_3 (°C)	LMTD (°C)	A (m²)
0	4	4.0	90	90	0,0	90	86	0.0
25	4	25.5	90	90	69.7	68.5	64.5	0.2
35	4	34.1	90	90	97,6	59,9	55,9	0,3
45	4	42,7	90	90	125,5	51,3	47,3	0,5
55	4	51,3	90	90	153,3	42,7	38,7	0,8
65	4	59,9	90	90	181,2	34,1	30,1	1,2

T_o, $T_{1'}$, T_1, T_2, T_3 refer to the stream temperatures shown in Figure 1a. $Q_{R,HTST}$ denotes the thermal power exchanged, while A represents the heat transfer area. The overall heat transfer coefficient (U) used in the calculation of the heat exchange area (A) was 5000 W/m^{2}K [36].

Table S4. Heat transfer and design calculations for the heat exchanger (*H*) used in the conventional thermal HTST pasteurization process of orange juice (Figure 1a), as a function of the waste heat recovery efficiency WHR.

WHR (%)	$T_{1'}$ (°C)	T_1 (°C)	$Q_{H,HTST}$ (kW)	S_{H1} (kg/h)	LMTD (°C)	A (m^2)
0	4.0	90	278.8	456.0	63.6	1.5
25	25.5	90	209.1	342.0	56.2	1.2
35	34.1	90	181.2	296.4	53.1	1.1
45	42.7	90	153.3	250.8	50.0	1.0
55	51.3	90	125.5	205.2	46.7	0.9
65	59.9	90	97.6	159.6	43.3	0.8

$T_{1'}$ and T_1 refer to the stream temperatures shown in Figure 1a. $Q_{H,HTST}$ and S_H denote the residual thermal power and steam consumption, respectively. A represents the heat exchange area of the heat exchanger. The overall heat transfer coefficient (U) used in the calculation of the heat exchange area (A) was 3000 W/m^{2}K [36].

Table S5. Heat transfer and design calculations for the cooling heat exchanger (*C1*) used in the conventional thermal HTST pasteurization process of orange juice (Figure 1a), as a function of the waste heat recovery efficiency WHR.

WHR (%)	T_3 (°C)	T_4 (°C)	Q_{C1} (kW)	T_{c1} (°C)	T_{c2} (°C)	W_{c1} (kg/h)	LMTD (°C)	A (m^2)
0	90	7	269.1	2	12	23172.5	26.6	2.0
25	68.5	7	199.4	2	12	17170.0	21.2	1.9
35	59.9	7	171.5	2	12	14769.0	19.0	1.8
45	51.3	7	143.6	2	12	12368.0	16.6	1.7
55	42.7	7	115.7	2	12	9967.0	14.2	1.6
65	34.1	7	87.8	2	12	7566.0	11.5	1.5

T_3 and T_4 refer to the stream temperatures shown in Figure 1a. T_{C1} and T_{C2} denote the inlet and outlet temperature of cold water. Q_{C1} and W_{c1} represent the thermal power and cooling water consumption, respectively. A represents the heat exchange area of the heat exchanger. The overall heat transfer coefficient (U) used in the calculation of the heat exchange area (A) was 5000 W/m^{2}K [36].

Calculation for heat exchanger of involved in the PEF system for pasteurization of orange juice (Figure 1b)

Table S6. Heat transfer and design calculations for the heat recovery exchanger (R) used in the PEF system for pasteurization of orange juice (Figure 1a), as a function of the waste heat recovery efficiency WHR.

WHR (%)	T_o (°C)	T_1 (°C)	$T_{1'}$ (°C)	$Q_{R,PEF}$ (kW)	T_2 (°C)	T_3 (°C)	LMTD (°C)	A (m²)
	4	35	4.0	0.0	47.5	47.5	43.5	0.0
	4	45	4.0	0.0	54.6	54.6	50.6	0.0
0	4	55	4.0	0.0	59.6	59.6	55.6	0.0
	4	35	11.8	25.1	47.5	39.75	35.8	0.1
	4	45	14.3	33.2	54.6	44.35	40.4	0.2
25	4	55	16.8	41.3	59.6	46.85	42.9	0.2
	4	35	14.9	35.2	47.5	36.65	32.7	0.2
	4	45	18.4	46.5	54.6	40.25	36.3	0.3
35	4	55	21.9	57.9	59.6	41.75	37.8	0.3
	4	35	18.0	45.2	47.5	33.55	29.6	0.3
	4	45	22.5	59.8	54.6	36.15	32.2	0.4
45	4	55	27.0	74.4	59.6	36.65	32.7	0.5
	4	35	21.1	55.3	47.5	30.45	26.5	0.4
	4	45	26.6	73.1	54.6	32.05	28.1	0.5
55	4	55	32.1	90.9	59.6	31.55	27.6	0.7
	4	35	24.2	65.3	47.5	27.35	23.4	0.6
	4	45	30.7	86.4	54.6	27.95	24.0	0.7
65	4	55	37.2	107.5	59.6	26.45	22.5	1.0

T_o, $T_{1'}$, T_1, T_2, T_3 refer to the stream temperatures shown in Figure 1b. $Q_{R,PEF}$ denotes the thermal power exchanged, while A represents the heat transfer area. The overall heat transfer coefficient (U) used in the calculation of the heat exchange area (A) was 5000 W/m²K [36].

Table S7. Heat transfer and design calculations for the heat exchanger (*H*) used in the PEF system for pasteurization of orange juice, as illustrated in the schematic of Figure 1a, as a function of the waste heat recovery efficiency WHR.

WHR (%)	$T_{1'}$ (°C)	T_1 (°C)	$Q_{H,PEF}$ (kW)	S_H (kg/h)	LMTD (°C)	A (m^2)
	4.0	35	100.5	160.3	79.5	0.4
	4.0	45	132.9	212.0	73.6	0.6
0	4.0	55	165.3	263.7	67.3	0.8
	11.75	35	75.4	120.2	76.0	0.3
	14.25	45	99.7	159.0	69.2	0.5
25	16.75	55	124.0	197.8	62.2	0.7
	14.85	35	65.3	104.2	74.6	0.3
	18.35	45	86.4	137.8	67.4	0.4
35	21.85	55	107.5	171.4	60.1	0.6
	17.95	35	55.3	88.2	73.2	0.3
	22.45	45	73.1	116.6	65.6	0.4
45	26.95	55	90.9	145.0	57.9	0.5
	21.05	35	45.2	72.1	71.7	0.2
	26.55	45	59.8	95.4	63.8	0.3
55	32.05	55	74.4	118.7	55.7	0.4
	24.15	35	35.2	56.1	70.3	0.2
	30.65	45	46.5	74.2	61.9	0.3
65	37.15	55	57.9	92.3	53.4	0.4

$T_{1'}$ and T_1 refer to the stream temperatures shown in Figure 1ba. $Q_{H,PEF}$ and S_H denote the residual thermal power and steam consumption, respectively. A represents the heat exchange area of the heat exchanger. The overall heat transfer coefficient (U) used in the calculation of the heat exchange area (A) was 3000 W/m^2K [36].

Table S8. Heat transfer and design calculations for the cooling heat exchanger (*C2*) used in the PEF system for pasteurization of orange juice, as illustrated in the schematic of Figure 1a, as a function of the waste heat recovery efficiency WHR.

WHR (%)	T_2 (°C)	T_1 (°C)	Q_{C2} (kW)	$T_{C2,1}$ (°C)	$T_{C2,2}$ (°C)	W_{C2} (kg/h)	LMTD (°C)	A (m^2)
	47.5	35	40.5	2	12	3489.8	34.2	0.2
	54.6	45	31.1	2	12	2680.2	42.8	0.1
0	59.6	55	14.9	2	12	1284.3	50.3	0.1
	47.5	35	40.5	2	12	3489.8	34.2	0.2
	54.6	45	31.1	2	12	2680.2	42.8	0.1
25	59.6	55	14.9	2	12	1284.3	50.3	0.1
	47.5	35	40.5	2	12	3489.8	34.2	0.2
	54.6	45	31.1	2	12	2680.2	42.8	0.1
35	59.6	55	14.9	2	12	1284.3	50.3	0.1
	47.5	35	40.5	2	12	3489.8	34.2	0.2
	54.6	45	31.1	2	12	2680.2	42.8	0.1
45	59.6	55	14.9	2	12	1284.3	50.3	0.1
	47.5	35	40.5	2	12	3489.8	34.2	0.2
	54.6	45	31.1	2	12	2680.2	42.8	0.1
55	59.6	55	14.9	2	12	1284.3	50.3	0.1
	47.5	35	40.5	2	12	3489.8	34.2	0.2
	54.6	45	31.1	2	12	2680.2	42.8	0.1
65	59.6	55	14.9	2	12	1284.3	50.3	0.1

T_2 and T_1 refer to the stream temperatures shown in Figure 1b. $T_{C2,1}$ and $T_{C2,2}$ denote the inlet and outlet temperatures of the cold water entering and leaving exchanger C2, respectively. Q_{C2} and W_{c2} represent the thermal power and cooling water consumption, respectively. A represents the heat exchange area of the heat exchanger.

Table S9. Heat transfer and design calculations for the cooling heat exchanger (*C1*) used in the PEF system for pasteurization of orange juice, as illustrated in the schematic of Figure 1a, as a function of the waste heat recovery efficiency WHR.

WHR (%)	T_3 (°C)	T_4 (°C)	Q_{C1} (kW)	$T_{C1,1}$ (°C)	$T_{C1,2}$ (°C)	W_{C2} (kg/h)	LMTD (°C)	A (m²)
	47.5	7	131.3	2	12	11307.1	15.6	1.7
	54.6	7	154.3	2	12	13289.3	17.6	1.8
0	59.6	7	170.5	2	12	14685.2	18.9	1.8
	39.8	7	106.2	2	12	9143.4	13.3	1.6
	44.4	7	121.1	2	12	10427.6	14.6	1.7
25	46.9	7	129.2	2	12	11125.6	15.4	1.7
	36.7	7	96.1	2	12	8277.9	12.3	1.6
	40.3	7	107.8	2	12	9283.0	13.4	1.6
35	41.8	7	112.6	2	12	9701.7	13.9	1.6
	33.6	7	86.1	2	12	7412.4	11.3	1.5
	36.2	7	94.5	2	12	8138.3	12.2	1.6
45	36.7	7	96.1	2	12	8277.9	12.3	1.6
	30.5	7	76.0	2	12	6546.9	10.3	1.5
	32.1	7	81.2	2	12	6993.6	10.8	1.5
55	31.6	7	79.6	2	12	6854.0	10.7	1.5
	27.4	7	66.0	2	12	5681.4	9.2	1.4
	28.0	7	67.9	2	12	5849.0	9.4	1.4
65	26.5	7	63.1	2	12	5430.2	8.9	1.4

T_3 and T_4 refer to the stream temperatures shown in Figure 1b. $T_{C1,1}$ and $T_{C1,2}$ denote the inlet and outlet temperatures of the cold water entering and leaving exchanger C1, respectively. Q_{C1} and W_{c1} represent the thermal power and cooling water consumption, respectively. A represents the heat exchange area of the heat exchanger.

3. Life cycle inventory

Table S10. Life cycle inventory analysis of resource consumption for pasteurizing one liter of orange juice by HTST and heat-assisted PEF process with a preheating temperature of 55°C and a recovery efficiency of 35%.

Resources	Unit	HTST	PEF
Electricity	kWh/L	0.069	0.054
Natural gas	m³/L	0.003	0.001
Water	m³/L	0.005	0.004

4. Assessment of Specific Thermal Energy Requirements, Temperature Profiles, and Water Consumption in PEF Pasteurization

Table S11. Specific thermal energy ($W_{th,PEF}$) values for the PEF pasteurization process supplied during preheating of orange juice at different inlet temperatures (T_1) to the PEF chambers as a function of the waste heat recovery efficiency (WHR).

Waste Heat Recovery Efficiency WHR (%)	T_1 (°C)	$W_{th,PEF}$ (kJ/kg)
0	35	120.6
	45	159.5
	55	198.4
25	35	90.4
	45	119.6
	55	148.8
35	35	78.4
	45	103.7
	55	129.0
45	35	66.3
	45	87.7
	55	109.1
55	35	54.3
	45	71.8
	55	89.3
65	35	42.2
	45	55.8
	55	69.4

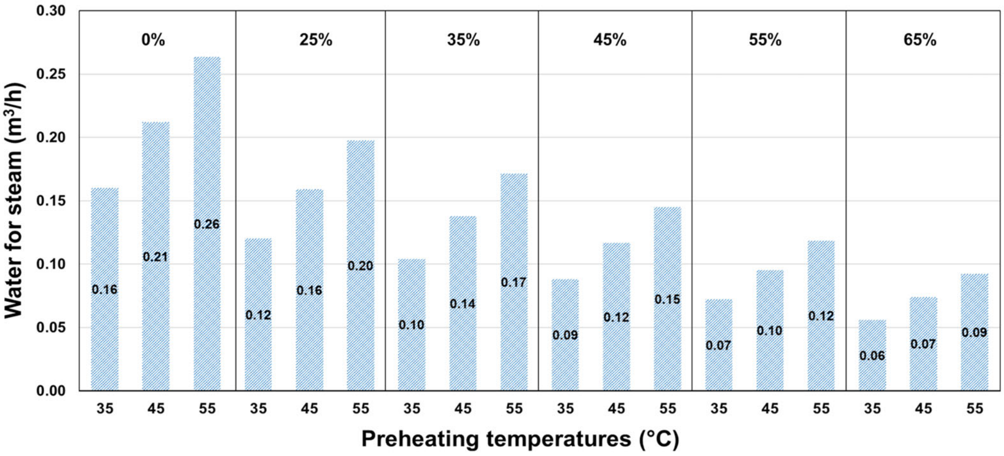

Figure S2. Water consumption for the steam generation during the PEF pasteurization process as a function of the waste heat recovery efficiency at different preheating temperatures T_1 of the orange juice.

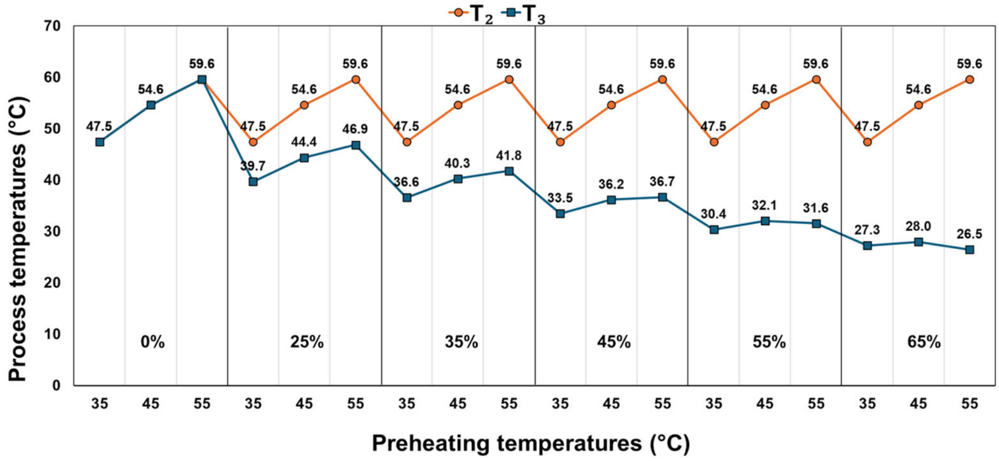

Figure S3. Temperature profiles of PEF-pasteurized orange juice during the thermal recovery step, with varying preheating temperature T_1 and waste heat recovery (WHR) efficiencies.

5. **Assessment of Net Present Value (NPV) for the PEF Pasteurization Process**

Table S12. Computed NPV values for the PEF pasteurization process at different combinations of preheating temperatures T_1 and waste heat recovery efficiency (WHR).

Waste Heat Recovery Efficiency WHR (%)	T_1 (°C)	NPV* (€)
0	35	921,747.28
	45	485,467.04
	55	248,501.19
25	35	674,688.57
	45	481,570.11
	55	454,773.90
35	35	335,293.96
	45	196,261.03
	55	223,550.28
45	35	-4,100.65
	45	-89,048.06
	55	-7,673.28
55	35	-343,495.26
	45	-374,357.15
	55	-238,896.85
65	35	-682,889.87
	45	-659,666.23
	55	-470,120.41

*The utility costs, including water, natural gas, and electricity, were computed using the following unit costs: 3.5 €/m^3 for water, 0.6 €/smc for natural gas, and 0.25 €/kWh for electricity, respectively.

6. Energy scenario

Table S13. Energy scenarios based on electricity and natural gas costs, with water costs fixed at 3.5 €/m^3.

Scenario	Electric Energy (€/kWh)	Natural Gas (€/smc)
1	0.25	0.6
2	0.25	1.5
3	0.35	0.6
4	0.35	1.5

7. Regional regulatory status of PEF-treated foods

foods MDPI

Table S14. Comparative regulatory frameworks for PEF-treated juices. 1

Region	Regulatory Authority	Classification	Approval Pathway	Labeling Requirements	Compliance Strategy	Ref.
United States (USA)	FDA	May require GRAS status or food additive petition	Scientific evidence; GRAS via expert consensus	No specific labeling	Submit safety data; consult GRAS panels; early FDA engagement	[63],[64], [68]
European Union (EU)	EFSA, EC	May be classified as "Novel Food" under Regulation (EU) 2015/2283	Requires prior authorization; must demonstrate safety, nutritional value, and no misleading of consumers	No specific mandate; general transparency	Submit Novel Food dossier; follow EFSA guidance	[65-66]
Codex Alimentarius	Codex Committee on Food Hygiene (FAO/WHO)	No specific classification for PEF	General food hygiene principles apply; not legally binding but internationally influential	No specific guidelines	Align with Codex for global harmonization	[69]
Canada	Health Canada	Novel if food properties are altered	Pre-market notification based on impact	No specific labeling	Determine novelty; prepare scientific justification	[70]
Australia & New Zealand	FSANZ	Case-by-case as novel technology	Case-by-case evaluation based on the extent of food modification	Not required unless PEF affects food identity	Early FSANZ engagement; assess structural changes	[70]
Asia (e.g., China, Japan)	Varies by country (e.g., NHC in China, MHLW in Japan)	Regulatory definitions not always clear	Generally limited precedent for PEF; approval pathways often undefined or product-specific	Varies by country	Monitor regulations; collaborate with regional experts	[71]

2
